# Backward bifurcation and hysteresis in models of recurrent tuberculosis

**DOI:** 10.1371/journal.pone.0194256

**Published:** 2018-03-22

**Authors:** Isaac Mwangi Wangari, Lewi Stone

**Affiliations:** 1 School of Science, Department of Mathematics and Geospatial Sciences, Royal Melbourne Institute of Technology, Melbourne, Victoria, Australia; 2 Biomathematics Unit, Department of Zoology, Faculty of Life Sciences, Tel Aviv University, Tel Aviv, Israel; Swiss Tropical and Public Health Institute, SWITZERLAND

## Abstract

An epidemiological model is presented that provides a comprehensive description of the transmission pathways involved for recurrent tuberculosis (TB), whereby cured individuals can become reinfected. Our main goal is to determine conditions that lead to the appearance of a backward bifurcation. This occurs when an asymptotically stable infection free equilibrium concurrently exists with a stable non-trivial equilibria even though the basic reproduction number *R*_0_ is less than unity. Although, some 10-30% cases of TB are recurrent, the role of recurrent TB as far as the formation of backward bifurcation is concerned, is rarely if ever studied. The model used here incorporates progressive primary infection, exogenous reinfection, endogenous reactivation and recurrent TB as transmission mechanisms that contribute to TB progression. Unlike other studies of TB dynamics that make use of frequency dependent transmission rates, our analysis provides exact backward bifurcation threshold conditions without resorting to commonly applied approximations and simplifying assumptions. Exploration of the model through analytical and numerical analysis reveal that recurrent TB is sometimes capable of triggering hysteresis effects which allow TB to persist when *R*_0_ < 1 even though there is no backward bifurcation. Furthermore, recurrent TB can independently induce backward bifurcation phenomena if it exceeds a certain threshold.

## Introduction

In 1882 *Mycobacterium tuberculosis* was identified by Robert Koch [1] as the aetiological agent responsible for tuberculosis (TB). Despite all attempts to control its spread by modern medical science, TB has become one of the most widespread and serious of all infectious diseases today [2]. The disease is transmitted from one person to another in tiny microscopic droplets when a person with pulmonary TB expels bacteria into the air by either through coughing, sneezing, singing, laughing or other related activities that involve airborne pathways. Amongst all infectious diseases, TB is one of the leading causes of death worldwide, and second only to human immunodeficiency virus (HIV) [3, 4]. Approximately a quarter of the global population harbours the TB bacteria and another eight to nine million new cases of tuberculosis emerge every year [5]. Extraordinarily, TB is a treatable disease and can be prevented and cured through the use of prophylaxis and therapeutics for individuals with latent and clinically active TB respectively. Such treatment should in theory be an effective strategy for controlling the spread of TB. However, it has failed in practice due to the inability to distribute sufficient drug treatment, usually in the form of antibiotics, to the world’s population combined with the difficulties of ensuring compliance to the required lengthy treatment program. Moreover, erratic treatment has led to the evolution of multi-drug resistant tuberculosis, giving rise to the fear that TB may become an untreatable disease in the not too distant future. These problems, taken together, have led the World Health Organization (WHO) to formulate a post-2015 global “Stop TB Strategy” [2] to “end the global TB epidemic.”

A TB episode may have an exogenous or endogenous origin. Exogenous refers to a disease episode that results from recent exposure to some external infectious source (typically, contact with an infectious person). Endogenous designates situations where the individual is already harbouring the causing agent, which is under some healthy control by the immune system, but which destabilizes and leads to disease. TB is unusual in that it can arise via both exogenous and endogenous infection. The pathogenesis of TB is characterized by the infection either remaining dormant, often for a long period that may last years, or progress directly to active TB where clinical symptoms immediately manifest. The latter process is referred to as fast primary progression. The particular course of the disease depends on the host’s immune response towards the tubercle bacilli. Thus, the exposure to tubercle bacilli does not necessarily result in the manifestation of clinical forms of TB. Studies suggest that only 5-10% of individuals progress directly to the active stage after exposure to bacilli [6, 7]. The other component of the population of exposed individuals develop dormant TB and may remain latently infected, possibly for the rest of their lifetime. However, destabilization of the immune system by the pathogen within the latently infected host can trigger endogenous reactivation, in which latent bacilli are reactivated and cause clinical *Mycobacterium tuberculosis*. The lifetime risk of a latently infected individual to progress to the infectious stage is approximately 5-10% [8].

We are particularly interested in recurrent TB, which is defined as the emergence of a second episode of TB after the first episode has been successfully cured [9]. This often arises following treatment, because an individual that recovers from a first episode of the disease does not necessarily gain permanent immunity to a second. Approximately 10-30% of all cases of TB are due to recurrent tuberculosis [10, 11], and multiple episodes are largely attributable to ineffective or poorly implemented tuberculosis control programs. Although recurrent TB is recognized as a serious problem it receives little attention [10]. Through the use of advanced molecular fingerprinting techniques, TB recurrence has been classified into two fundamental forms of infection: i) relapse of the original infecting strain, and ii) reinfection with a new strain of *Mycobacterium tuberculosis*. The role of reinfection and relapse to the overall burden of tuberculosis recurrence is not well understood and this has potential public health implications [12]. It is important to note that the system adopted by the World Health Organization in recording and reporting TB cases does not differentiate between true relapse (i.e., reactivation of latent TB) and reinfection (exogenous acquisition of TB) and classifies as relapse any recurrence of TB [11]. This greatly affects the collection and analysis of data making it even more difficult to assess the specific role of reinfection.

In [13] it was found that persons who had TB have a greatly increased risk of developing TB when reinfected. Moreover, the study suggests that the incidence rate of TB attributable to reinfection after successful treatment is four times higher than that of new TB [13]. A key goal of this paper is to investigate the effect of recurrent TB on the formation of backward bifurcations through detailed mathematical modelling. To our knowledge, no other study has examined this possibility.

**Fig 1:**
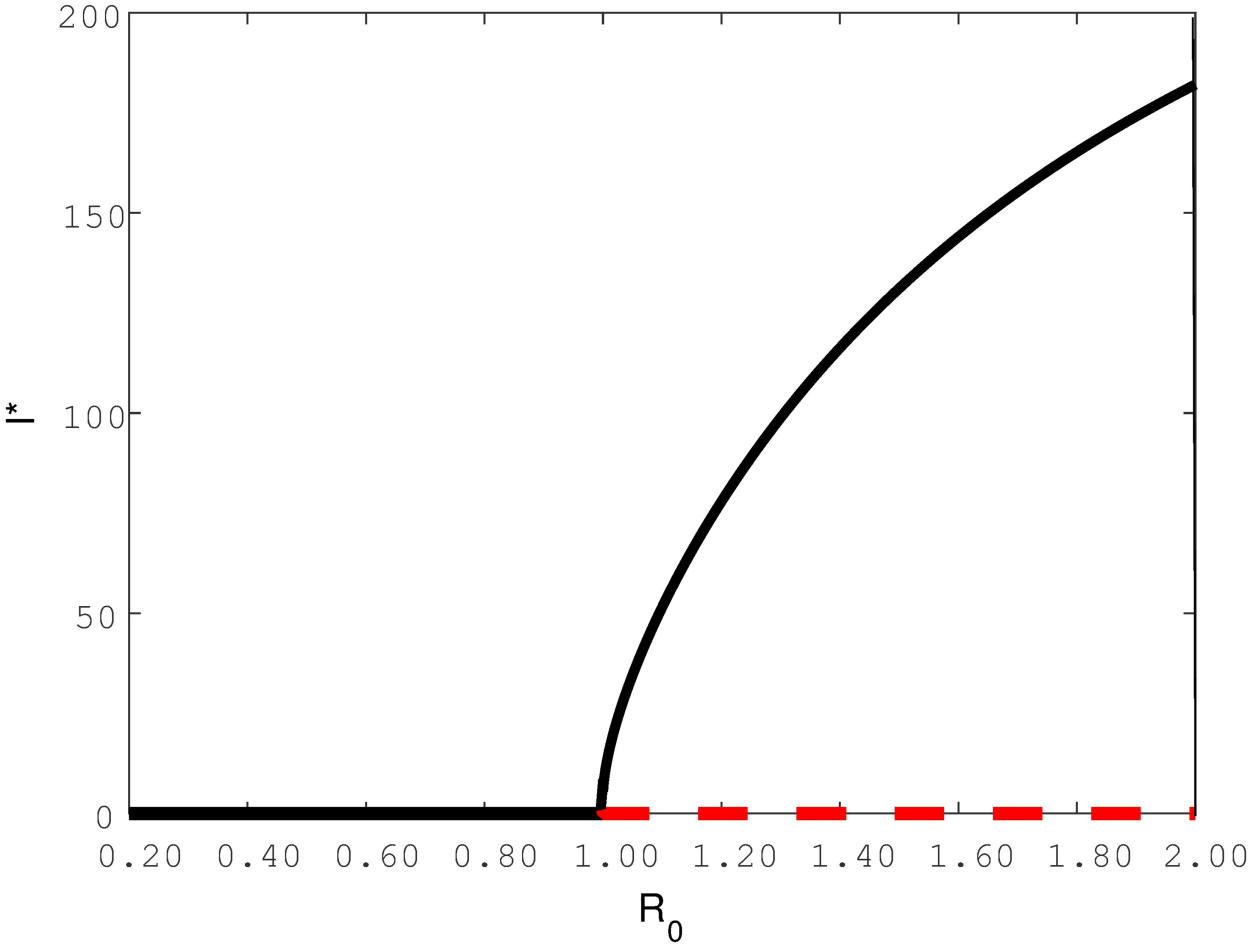
Qualitative illustration of forward bifurcation by plotting I* versus *R*_0_. The red dotted line represent unstable equilibria while the black solid line represent stable equilibria.

Similar to other infectious diseases, the ability for TB to persist in a population or go extinct is to a large degree governed by the basic reproduction number, *R*_0_, which is defined as the number of secondary cases an infected individual will generate when introduced into a completely susceptible population [14–17]. This fundamental epidemiological quantity determines whether an infection will be able, on average, to reproduce itself (*R*_0_ > 1) in the population or not (*R*_0_ < 1). Characteristically, when *R*_0_ is below unity, the introduction of a few infected individuals in a susceptible population will only lead to disease die-out, as the disease is unable to reproduce itself or transmit through the population effectively. Conversely, when *R*_0_ is above unity an epidemic may trigger and long-term disease persistence is feasible. Classical epidemic models therefore often have two intrinsic equilibria: a disease free equilibrium (DFE) and a non-trivial endemic equilibrium. By endemic is meant an equilibrium in which the number of infectives is greater than zero. The stability of these equilibria switch at the (transcritical) bifurcation point when *R*_0_ = 1. Fig 1 illustrates the more typical forward bifurcation by plotting the equilibrium number of infectives (I*) in a population as a function of *R*_0_. This shows that a stable DFE in which I* = 0 exists when *R*_0_ < 1. However, when crossing the threshold to a regime where *R*_0_ > 1, there is a change of stability where the DFE becomes unstable while the previously unstable endemic equilibrium stabilizes.

### Backward bifurcations

**Fig 2:**
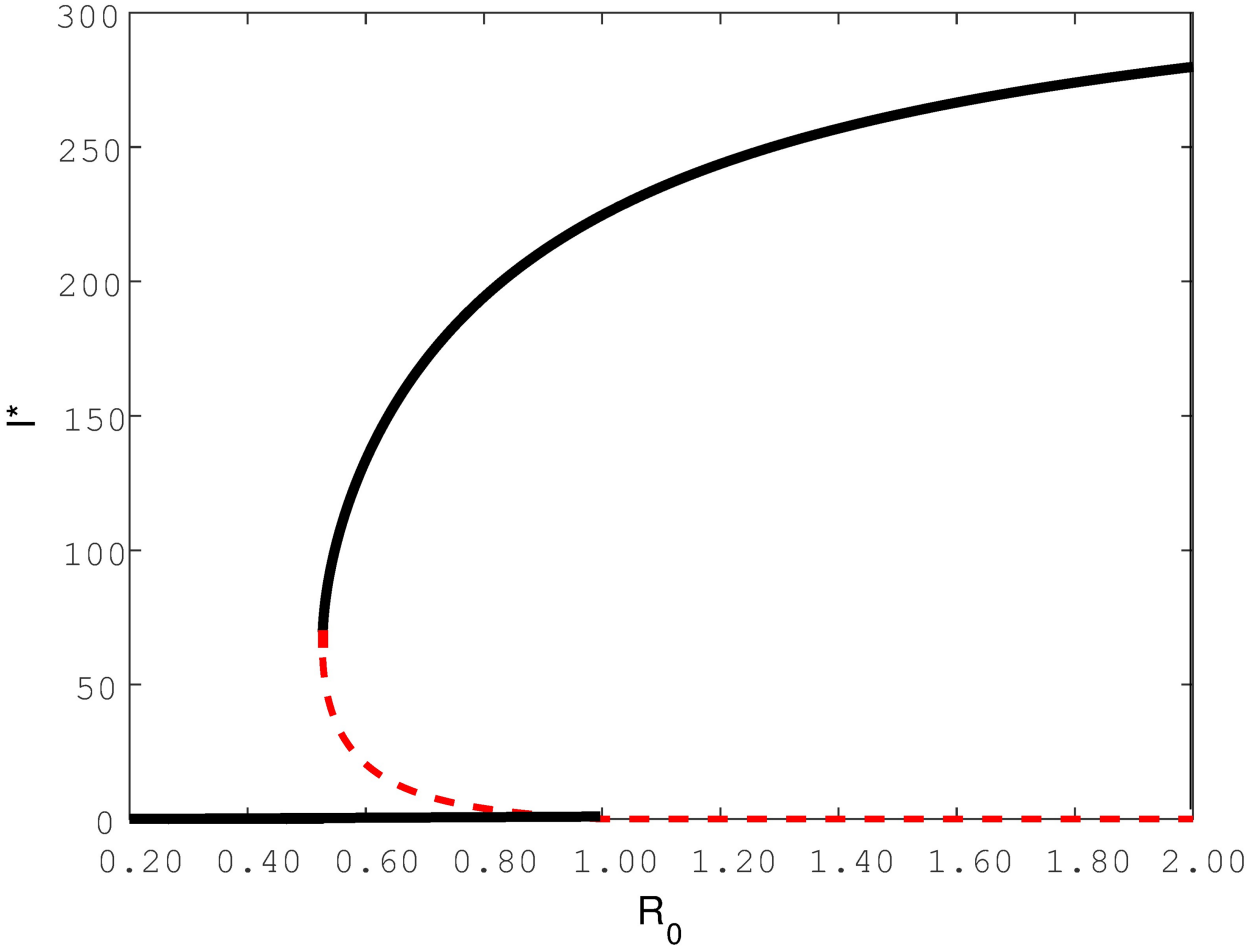
Qualitative illustration of backward bifurcation at *R*_0_ = 1. The critical value of *R*_0_, namely *R*_0_ = *R*_*c*_ = 0.53. The red dotted line represent unstable equilibria while the black solid line represent stable equilibria.

For decades, it has been widely accepted that the condition *R*_0_ < 1 is an essential requirement for the elimination of a disease. However, this viewpoint has been recently challenged. Instead, the phenomenon of *backward bifurcation* offers a different interpretation since it shows that although *R*_0_ < 1 and the DFE is stable, there might still be another stable endemic equilibrium coexisting simultaneously. Thus even though *R*_0_ < 1, a population may still reside at an endemic equilibrium at which the disease persists indefinitely. When there are multiple stable equilibria coexisting simultaneously, the final equilibrium a population will reach is dependent on the initial conditions of its sub-populations. Fig 2 provides a typical bifurcation diagram that shows the key features of a backward bifurcation. Note that three equilibria coexist when *R*_0_ is in the range 0 < *R*_*c*_ < *R*_0_ < 1, where *R*_*c*_ is a critical value, which in Fig 2 is *R*_*c*_ = 0.53. In this range, the “middle” equilibrium is unstable, while the other two outer equilibria (the DFE and the endemic equilibrium) are both stable. When *R*_0_ < *R*_*c*_, only the DFE equilibrium exists and is stable.

Multiple coexisting equilibria can lead to interesting dynamics when, for example *R*_0_ varies slowly as shown in Fig 2. Suppose, for example, that the infective population is close to extinction I* = 0 (the DFE) and *R*_0_ increases slowly. As soon as *R*_0_ passes through the threshold point *R*_0_ = 1 the number of infectives will suddenly jump from close to I* = 0 to the large endemic equilibrium (I* > 0), (see Fig 2). Similarly, when the infective population sits close to the endemic equilibrium and *R*_0_ reduces slowly through the threshold point *R*_0_ = 1, rather than switching to the DFE, the infectives remain close and converge to the stable endemic equilibrium. However, as soon as *R*_0_ reduces below the threshold *R*_*c*_, the infective population immediately jumps towards zero (the DFE), while the endemic equilibrium disappears.

Backward bifurcations have major implications for infectious diseases such as TB, since control programs based on reducing *R*_0_ below unity may be ineffective given the disease might be able to easily persist indefinitely under such conditions. Certainly this non-intuitive possibility will have to be taken into account if the new WHO “Stop TB Strategy” is to succeed. Lipsitch and Murray [18] considered the phenomenon of backward bifurcation caused by exogenous reinfection in TB epidemic models with skepticism and suggested that it is unlikely to occur for realistic parameter values. However, TB epidemiology in the last two decades has greatly changed and challenges Lipsitch and Murray [18] line of argument. More recent research supports the notion that individuals experience multiple infections throughout the course of their life as a result of reinfection, especially in high-incidence TB areas [19–21], all conditions which could promote the existence of backward bifurcations. More on this controversy will be elaborated in the text.

## Model of recurrent tuberculosis

Although, there are numerous TB models that have attempted to include the recurrent TB pathway (for instance see [22–27]), none have explored its role in the formation of backward bifurcation. Yang et al. [24] investigated the impact of multiple infections and long latency on the dynamics of recurrent tuberculosis. Their results suggest that a backward bifurcation is expected to occur when a critical value of the disease incubation period is exceeded. However, Yang et al. [24] assume that the reinfection of recovered individuals is negligible, arguing that such a pathway increases non-linearity and makes the model mathematically intractable. The models of Feng et al. [23] and Kar et al. [22] were all based on the assumption that individuals pass through a long latency period before TB reactivated to clinically active TB. However, based on the natural history of TB, fast primary progression of TB forms an important process through which symptomatic TB emerges [28] and needs to be included. Indeed, Feng et al. [23] incorporated a particular recurrent TB pathway in their model; however, their simplifying assumptions hindered further exploration with regard to its role in causing a backward bifurcation. The models developed by Gomes et al. [25] and Herrera et al. [26] attempted to incorporate exogenous re-infection, partial immunity to reinfection and primary progression. However, neither group examined how recurrent TB reinfection pathways could lead to a backward bifurcation. Instead their main objective was to study the reinfection threshold. Hence, our main aim is to study reinfection amongst recovered individuals and deduce the epidemiological implication it has especially with regards to the possible formation of a backward bifurcation. For this purpose we develop a TB model that includes fast primary progression and possible reinfection pathways.

**Fig 3:**
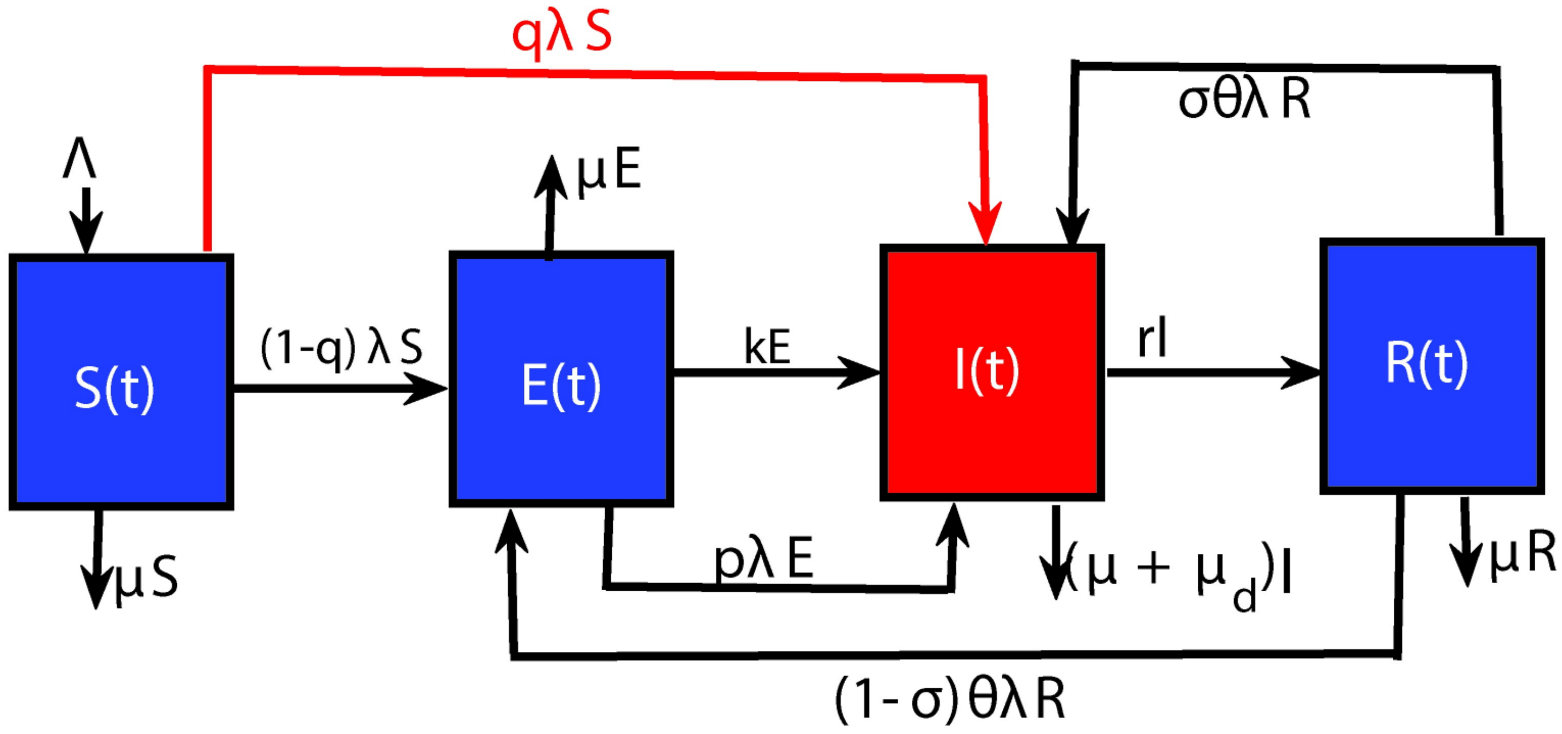
Schematic diagram of the main processes involved in TB infection according to model Eq (1).

The model is represented graphically in Fig 3 and is based on the following processes. We assume that every individual in the population belongs to one of four broad classes: susceptible individuals (*S*), exposed individuals (*E*) (these are infected individuals who are not able to transmit infection), individuals with active TB (*I*) (who manifests symptoms and are able to pass on the infection), and recovered/treated individuals (*R*). Individuals have the potential to move through these four classes, as for example in the loop *S* → *E* → *I* → *R* → *I* or *S* → *E* → *I* → *R* → *E*, upon contact with the disease.

Susceptible individuals are generated by recruitment through births and immigration at a rate Λ. Upon contact with an infective, a small proportion *q* of infected susceptible individuals follow the fast primary progression route (i.e they move directly to the infective class) while the rest (1 − *q*) move to the exposed class where they pass through a long latency period before reactivation and becoming infectious. Infected individuals who recover from the disease then move to the recovered class.

The model includes three different pathways for exogenous reinfection as shown in Fig 3:

(i) **Path** **A** = *pλE*, where exposed individuals, who have partial immunity against exogenous reinfection (See [23]), become reinfected and progress to active TB,
(ii) **Path** **B** = (1 − *σ*)*θ*λ*R*, where recovered individuals become reinfected and progress to the exposed sub-population and
(iii) **Path** **C** = *σθ*λ*R*, where recovered individuals become reinfected and progress to active TB.

In our model, the parameter *p* measures the degree of partial protection against TB among latently infected individuals and *p*λ*E* is the exogenous reinfection incidence rate. *σ* measures the probability of fast progression to the infectious class after reinfection. Following the studies of [22, 23, 25, 26, 29], *p* = 1 implies that the body does not render protection against exogenous reinfection while 0 < *p* < 1 implies the body is partially immune against exogenous reinfection (i.e latent infection provides immunity against new infections) [27, 30]. Note that *p* > 1 would imply that an individual with latent TB infection has increased susceptibility to become newly infected, as compared to the susceptibility of the general population [27]. This relates to studies which have found that recovered individuals are more likely to be susceptible to future TB infection than TB-naive individuals [13].

The parameter *θ* (0 < *θ* < 1) quantifies the amount of exogenous reinfection among TB recovered individuals via paths *B* and *C* while *σ* represent the probability of fast progression after reinfection amongst recovered individuals. *θ* < 1 indicates that recovered individuals have acquired some degree of partial protective immunity to TB, while *θ* > 1 indicates increased susceptibility.

The full model equation is given in terms of the rates of change of each of the sub-populations *S*, *E*, *I*, and *R* namely:

$$\begin{array}{ccc} \frac{dS}{dt} & = & \Lambda -\lambda S-\mu S,\\ \frac{dE}{dt} & = & (1-q)\lambda S+(1-\sigma )\theta \lambda R-p\lambda E-(k+\mu )E,\\ \frac{dI}{dt} & = & q\lambda S+\sigma \theta \lambda R+p\lambda E+kE-(\mu +r+\mu_{d})I,\\ \frac{dR}{dt} & = & rI-\theta \lambda R-\mu R. \end{array}$$ (1)

Note that the total population at time *t* is given by *N*(*t*),

$$\begin{array}{c} N(t)=S(t)+E(t)+I(t)+R(t). \end{array}$$

The model assumes a frequency dependent incidence rate. Here we have followed the convention of working with the so-called ‘force of infection’ λ defined as

$$\begin{array}{c} \lambda =\frac{c\beta I}{N} \end{array}$$ (2)

where *c* represents the host-host contact. The parameter *β* is the probability of a contact being infectious [31] and *I*/*N* denotes the likelihood that the encounter is with an individual with active TB (assuming random mixing) [32]. This form of incidence is considered to be more appropriate for infections in human populations [33]. That is individuals become infected when they come into contact with infected individuals regardless of the size of the human population [33, 34]. It is important to note that although a large number of previous TB models utilised frequency dependence incidence rates, most assume that the disease-induced death (*μ_d_*) is negligible for the purpose of mathematical simplification. Indeed the analysis becomes mathematically difficult or even intractable without making such assumptions, since otherwise the total population of the model *N*(*t*) might never remain constant. Both Feng et al. [23] and Porco et al. [7] resorted to this assumption to ensure that the total population *N*(*t*) remained constant, even though this was not true of their more general model formulation. In the analysis here, we make no such assumptions or simplifications and consider the total population to be varying in time.

In more detail, as Eq (1) shows, susceptible individuals move to the latently infected class *E* upon an effective contact with an infected individuals (*I*) at a rate λ*S*. The exposed sub-population (*E*) increases with the infection of susceptible individuals at a rate (1 − *q*)λ*S* and reinfection (recurrent TB) of recovered individuals at a rate (1 − *σ*)*θ*λ*R*. It decreases by exogenous re-infection (*p*λ*EI*), and endogenous reactivation (*kE*) upon which exposed individuals move to the infectious class. The infected sub-population is generated by fast primary progression of TB susceptibles (*q*λ*S*), exogenous reinfection amongst exposed sub-population (at rate *p*λ*E*), exogenous reinfection of recovered individuals (*σθ*λ*R*) and endogenous reactivation (*k*). The sub-population is decreased by per-capita recovery due to treatment *r*, and by disease induced death at rate *μ*_*d*_. Finally the recovered sub-population (*R*) is generated by the recovery of infected individuals and exogenous reinfection (at rate *θ*λ*R*). Note that we model natural death which affects all classes of the population at the same rate via the mortality parameter *μ*.

Our model assumes all immigrants are susceptible. While this excludes the realistic possibility of immigration of infectives (either latent individuals or individuals with active TB), it helps untangle the conditions that result in backward bifurcation. Previous modelling studies have already demonstrated that the immigration of individuals with latent TB or active TB are pathways that can trigger backward bifurcations [35, 36]. Progression to active TB is not uniform, as some infected individuals are more likely to progress to active TB than others. A number of models incorporating long and variable rate of progression have been constructed and analysed (for instance see [23, 37, 38]). Feng et al. [39] investigated the impact of variability in latency using arbitrary continuous distributions and found that such generalization did not result in qualitative differences in terms of the model dynamics. Before the Feng et al. [23] study, Blower et al. [37] formulated a differential equation model with two latent cohorts: one cohort consisted of those who rapidly develop TB after primary infection, while the second cohort involved individuals who develop the infection slowly through endogenous reactivation. Feng et al. [39] showed that the artificial divisions (as in Blower et al. [37]) play no role in the qualitative dynamics.

We also chose a single latent compartment over two latent compartments (fast and slow TB progression) for the purpose of mathematical tractability. The increased number of reinfection pathways needed with two compartments would add a considerable degree of model complexity, and become very difficult to analyse. Moreover, the original study of [23], where the role of reinfection in inducing backward bifurcation was first identified, consisted of a single latent compartment. The paper by [18] that criticised Feng et al. [23] also had a single latent compartment. Since our goal is to investigate how recurrent TB (reinfection of recovered individuals) can impact the backward bifurcation phenomenon found by Feng et al. [23], to keep the same single latent compartment structure.

Table (1) provides a detailed list and description of model parameters as well as typical parameter values used here, as obtained from the literature.

**Table 1:** Description of variables and parameters of model (1).

Variable	Interpretation			
S	Susceptible sub-population			
E	Asymptomatic and non-infectious individuals			
I	Symptomatic and infectious individuals			
R	Recovered individuals			
Parameter	Interpretation	Nominal value	Sources	Unit
*μ*	Natural death rate	0.016	[40]	*year*^−1^
*μ*_*d*_	Disease induced death rate	0.1	[23]	*year*^−1^
*θ*	Recurrent TB due to reinfection	0.25[0, 7.79]	[13, 27, 41]	*year*^−1^
*σ*	probability of fast progression after reinfection	0.05-0.20	[27]	*year*^−1^
*r*	per-capita recovery rate	2 (see S5 Appendix)	[23, 42]	*year*^−1^
*p*	Exogeneous re-infection	0.25[0, 1]	[23, 40, 43]	*year*^−1^
*q*	Primary progression rate	0.05	[7, 22, 23] [30, 37, 40]	*year*^−1^
*k*	Endogeneous reactivation rate	0.0002	[27, 41, 44]	*year*^−1^
*β*	Probability of becoming infected per contact	[0, 1]	− −	*year*^−1^
*c*	Mean number of contacts	variable	− −	*year*^−1^
Λ	Recruitment rate	100	[45]	*year*^−1^

## Model analysis

### Basic properties

Following the methods in Garba et al. [46], it is not difficult to prove that when all model parameters are nonnegative the state variables *S*(*t*), *I*(*t*), *E*(*t*) and *R*(*t*) are all positive for all time *t*. The equilibria of the model are found by setting the rates of all variables in the left-hand side of Eq (1) to zero. Clearly the equations have an intrinsic disease free equilibrium (DFE) given by $\left(\bar{S},\bar{E},\bar{I},\bar{R}\right)=P_{0}=(\frac{\Lambda }{\mu },0,0,0)$. The stability of the DFE is controlled by the basic reproduction number *R*_0_ which represents the average number of new infections generated by an infected individual when introduced into an entirely susceptible population. *R*_0_ may be determined using the next generation operator method (see [47]) as shown in S1 Appendix, where it is found that:

$$\begin{array}{c} R_{0}=\frac{c\beta (k+q\mu )}{\left(\mu +k\right)\left(\mu +r+\mu_{d}\right)}. \end{array}$$ (3)

It is possible to decouple the expression for *R*_0_ to account for slow TB progression and fast primary progression

$$\begin{array}{c} R_{0}=\underset{\text{Slow}\;\text{TB}\;R_{0}}{\underset{︸}{\left(\left(1-q\right)\frac{c\beta }{\mu +r+\mu_{d}}\right)\left(\frac{k}{\mu +k}\right)}}+\underset{\text{Fast}\;\text{TB}\;R_{0}}{\underset{︸}{\left(q\frac{c\beta }{\mu +r+\mu_{d}}\right)}}. \end{array}$$

The slow TB component of *R*_0_ can be obtained by observing that the average infectious period is given as $\frac{1}{\mu +r+\mu_{d}}$ and the probability of progressing from latent compartment to infective class is given as $(\frac{k}{\mu +k}).$ The average time an individual who starts in the latent compartment is expected to spend in the infectious compartment is, $\left(\frac{1}{\mu +r+\mu_{d}})\times (\frac{k}{\mu +k}\right).$ Multiplying this average time by (**1** − **q**)**c***β* yields the slow TB *R*_0_. Moreover, multiplying mean infectious period $\left(\frac{1}{\mu +r+\mu_{d}}\right)$ with *qcβ* yields the fast TB component of *R*_0_.

An important result is the following Theorem:

**Theorem 1** *Provided R*_0_ < 1, *the DFE of the model* (1) *is locally asymptotically stable, otherwise it is unstable*.

This general result has been reviewed in [48], and hence we do not prove Theorem 1 here. The theorem implies that it is possible to eradicate the disease from the community when *R*_0_ < 1 if the initial sizes of the sub-populations of model (1) are in the basin of attraction of the disease free equilibrium.

Interestingly, the formula for the basic reproduction number Eq (3) does not include the reinfection parameters *p* and *θ* despite the fact that these terms should contribute significantly to the emergence of new cases of TB infection. Hence, this already suggests that *R*_0_ alone is unable to completely quantify some key dynamical features of the TB epidemic, and is in fact the first sign that a backward bifurcation might be involved. It will emerge that the reinfection parameters *p* and *θ* play an important role and are responsible for the presence of the backward bifurcation intrinsic to this model.

### Backward bifurcation analysis

We begin by identifying the model’s endemic equilibrium points being mindful that there may in fact be several such points coexisting simultaneously. As before, to find the endemic equilibria (S*, E*, I*, R*) we set the rate Eq (1) to zero and solve for the equilibrium quantities S*, E*, I* and R* in terms of the force of infection λ (See S3 Appendix). Substituting these equilibrium quantities into the force of infection Eq (2) yields

$$\begin{array}{c} P\left(\lambda \right)=\lambda \left(a_{3}\lambda^{3}+a_{2}\lambda^{2}+a_{1}\lambda +a_{0}\right)=0, \end{array}$$ (4)

where

$$\begin{array}{c} a_{3}=\theta p,\;a_{2}=c\theta p\left(\beta_{0}-\beta \right),\;a_{1}=c\left[q\theta \mu +\theta k+\mu p\right](\beta_{1}-\beta ),\\ a_{0}=c\mu \left(k+\mu q\right)\left(\beta_{R}-\beta \right). \end{array}$$ (5)

and

$$\begin{array}{cc} \beta_{0} & =\frac{\theta p\left(\mu +\mu_{d}\right)+\left(1-q\right)\left(\mu +\mu_{d}\right)\theta +\left(1-\sigma \right)\theta r+\theta \left(k+\mu q\right)+p\left(\mu +r\right)}{c\theta p}, \end{array}$$ (6)

$$\begin{array}{c} \beta_{1}=\frac{\theta \left(\mu +\mu_{d}\right)\left(\mu +k\right)+\mu p\left(\mu +r+\mu_{d}\right)+r\theta \mu \left(1-\sigma \right)+\left(1-q\right)\left(\mu +\mu_{d}\right)\mu +r\left(\mu +k\right)+\mu \left(k+\mu q\right)}{c(q\theta \mu +\theta k+\mu p)}, \end{array}$$ (7)

$$\begin{array}{c} \beta_{R}=\frac{\left(\mu +r+\mu_{d}\right)\left(\mu +k\right)}{c(k+\mu q)}. \end{array}$$ (8)

One notes from Eq 4 that the reproductive number $R_{0}=\frac{\beta }{\beta_{R}}$, and also that the root λ = 0 corresponds to the DFE, where I* = 0. Now the roots of the cubic equation

$$\begin{array}{c} P_{1}\left(\lambda \right)=a_{3}\lambda^{3}+a_{2}\lambda^{2}+a_{1}\lambda +a_{0}=0 \end{array}$$ (9)

substituted in S*, E*, I*, R* yield the endemic equilibrium for any specific set of model parameters (see S3 Appendix).

### No recurrent TB (i.e *θ* = 0): Quadratic *P*_2_(λ)

We first examine the important particular parameter subset in which *σ* = *θ* = 0, that is, in the absence of recovered individuals becoming reinfected. This model has the same basic reproduction number as for the case *σ*, *θ* > 0, as *σ* and *θ* do not appear in the *R*_0_ expression (3). For *σ* = *θ* = 0 the third degree polynomial Eq (9) collapses to the quadratic:

$$\begin{array}{c} P_{2}\left(\lambda \right)=c_{2}\lambda^{2}+c_{1}\lambda +c_{0}=0, \end{array}$$ (10)

where

$$\begin{array}{c} c_{2}=\left(\mu +r\right)p,\;c_{1}=c\mu p\left(\bar{\beta_{1}}-\beta \right),\;c_{0}=c\mu \left(k+\mu q\right)\left(\beta_{R}-\beta \right),\;\\ \bar{\beta_{1}}=\beta_{1}{|}_{\sigma =\theta =0}=\frac{\left(\mu +r+\mu_{d}\right)\mu p+\left(\mu +k\right)\left(\mu +r\right)+\mu \mu_{d}\left(1-q\right)}{c\mu p}. \end{array}$$

In the above, we see that *c*_0_ < 0 corresponds to *R*_0_ > 1 and vice versa. Thus, in the absence of recurrent TB we deduce *c*_1_ < 0, *R*_0_ < 1 and $c_{1}^{2}-4c_{2}c_{0}>0$ (see Theorem 1 in S1 Appendix) which indicates conditions for the existence of a backward bifurcation, based on the roots of the quadratic Eq (10).

Note that Case (iii) of Theorem 1 in S1 Appendix stipulates the condition that $△=c_{1}^{2}-4c_{2}c_{0}>0$ which means there are two real positive endemic equilibria as required for a backward bifurcation to appear. In fact △ = 0 provides the critical point for the backward bifurcation where the two positive endemic equilibria collide and annihilate each other leaving the DFE as the only equilibria.

By setting △ = 0 we can determine the critical value of the transmission coefficient denoted by *β*_*c*_. For mathematical convenience let

$$\begin{array}{c} c_{1}=\phi_{1}-\phi_{2}\beta ,c_{0}=\phi_{3}-\phi_{4}\beta ,\;\phi_{1}=\left(\mu +r+\mu_{d}\right)\mu p+\left(\mu +k\right)\left(\mu +r\right)+\mu \mu_{d}\left(1-q\right),\;\\ \phi_{2}=c\mu p,\;\;\phi_{3}=\left(\mu +r+\mu_{d}\right)\left(\mu +k\right),\;\phi_{4}=c\mu \left(k+\mu q\right). \end{array}$$

Now the discriminant △(*β*) may be expressed in terms of *β*. Let *β*_*c*_ be the critical value of *β* for which the discriminant equals zero i.e.,

$$\begin{array}{c} △\left(\beta_{c}\right)=\phi_{2}^{2}\beta_{c}^{2}+2\left(2c_{2}\phi_{4}-\phi_{1}\phi_{2}\right)\beta_{c}+\left(\phi_{1}^{2}-4c_{2}\phi_{3}\right)=0. \end{array}$$

Some algebraic rearrangement gives

$$\begin{array}{c} \beta_{c}=\frac{\left(\phi_{1}\phi_{2}-2c_{2}\phi_{4}\right)+2\sqrt{c_{2}^{2}\phi_{4}^{2}+c_{2}\phi_{2}\left(\phi_{2}\phi_{3}-\phi_{1}\phi_{4}\right)}}{\phi_{2}^{2}}. \end{array}$$ (11)

The critical value of basic reproduction number denoted by *R*_*c*_ is obtained by replacing parameter *β* in *R*_0_ with *β*_*c*_ which yields

$$\begin{array}{c} R_{c}=(\frac{c(k+\mu q)}{\left(\mu +k\right)\left(\mu +r+\mu_{d}\right)})(\frac{\left(\phi_{1}\phi_{2}-2c_{2}\phi_{4}\right)+2\sqrt{c_{2}^{2}\phi_{4}^{2}+c_{2}\phi_{2}\left(\phi_{2}\phi_{3}-\phi_{1}\phi_{4}\right)}}{\phi_{2}^{2}}), \end{array}$$ (12)

where the right-hand term in large brackets is just *β*_*c*_.

**Fig 4:**
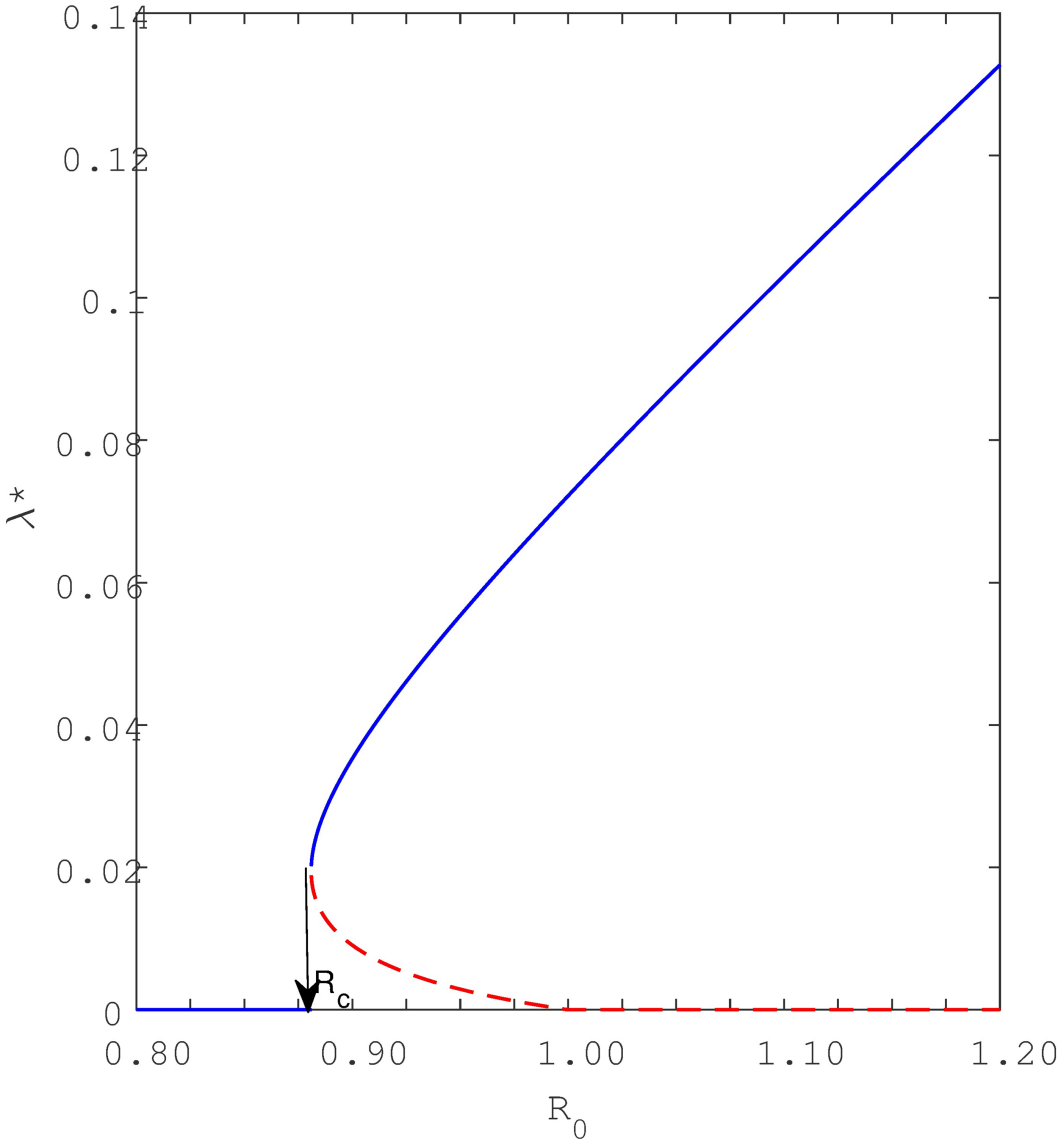
Illustration of backward bifurcation when there is no recurrent TB (i.e. *θ* = 0). Parameters are defined in Table (1) except *p* = 0.09 > *p*_*c*_ = 0.0658, *k* = 0.0002, *q* = 0.05, *c* = 60, *β* ∈ {0.4, 0.7} and *β*_*c*_ = 0.5099 corresponding to *R*_*c*_ = 0.8852. The blue solid line represent the stable equilibria while the red dotted line represent unstable equilibria.

In fact *R*_*c*_ defines a sub-threshold domain of bistable equilibria of the model system (1) in the sense that within the region *R*_*c*_ < *R*_0_ < 1 the model Eq (1) has two positive endemic equilibria simultaneously existing with a stable disease free equilibrium. Thus, the backward bifurcation for Eq (1) occurs for values of the basic reproduction number *R*_0_ that lie between *R*_*c*_ < *R*_0_ < 1. The associated backward bifurcation for the model without the reinfection pathways *A* and *B* (i.e, *σ* = *θ* = 0) shown in Fig 4 is obtained by plotting λ as a function of *β*. Fig 4 shows that model (1) has a disease free equilibrium which corresponds to λ = 0 and two non-trivial endemic equilibria which, according to numerical simulations, one is locally asymptotically stable (LAS) and the other is unstable (saddle). Shortly we will apply center manifold to examine stability and confirm coexistence of these three equilibria (see S2 Appendix). We thus find coexistence of two positive equilibria when *R*_0_ < 1, hence confirming that the model exhibits the phenomenon of backward bifurcation for *R*_*c*_ < *R*_0_ < 1.

**Fig 5:**
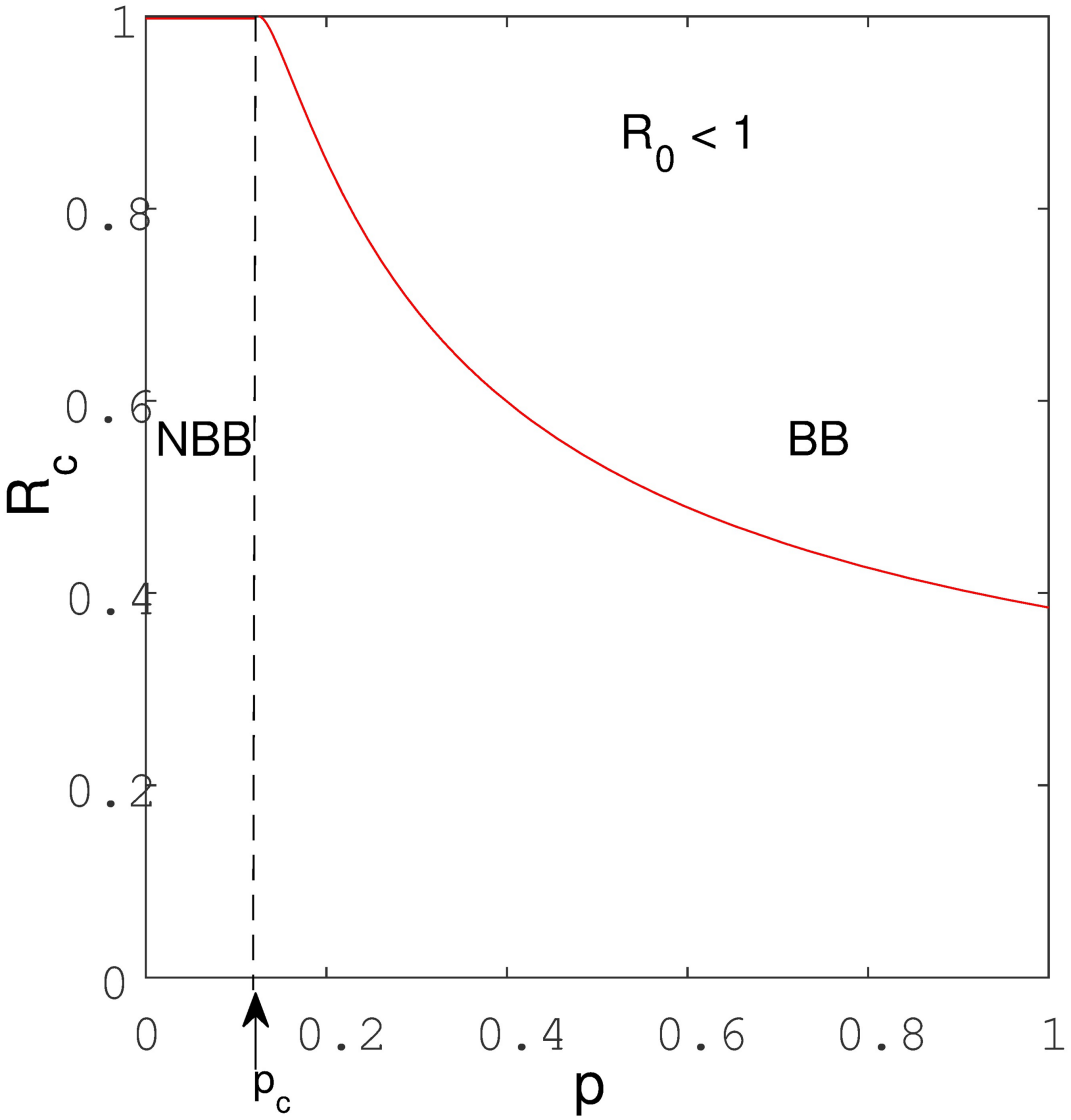
A plot of the critical value of *R*_*c*_ as a function of the level of exogenous reinfection (p). NBB and BB respectively denote no backward bifurcation and backward bifurcation regions. In the region denoted by NBB, the level of exogenous reinfection is too low to induce backward bifurcation while in region denoted by BB the level of exogenous reinfection is sufficient to cause multiple equilibria. Parameters used remain as shown in Table (1).

It can be seen from Eqs (11) and (12), that when exogenous reinfection parameter *p* increases, *R*_*c*_ decreases, and vice-versa. Hence, we can deduce from expression (12) that *R*_*c*_ is inversely proportional to the level of exogenous reinfection *p*. This observation is confirmed by Fig 5 which illustrates the effect of increasing exogenous reinfection *p* on *R*_*c*_. That is, with low values of exogenous reinfection the critical value *R*_*c*_ is high implying that the extent of the backward bifurcation regime becomes smaller as *R*_*c*_ becomes closer to unity (*R*_0_ = 1). The threshold implies that TB can be eliminated from the community if the basic reproduction number is maintained below *R*_*c*_ (*i*.*e R*_0_ < *R*_*c*_). More formally we have the following lemma:

**Lemma 1** *For model* Eq (1), *when* *σ* = *θ* = 0,

*(i) If R*_0_ > 1 *the model has one positive endemic equilibrium point*,
*(ii) If R*_*c*_ < *R*_0_ < 1 *the model has two positive endemic equilibria*,
*(iii) If R*_0_ < *R*_*c*_ *has only disease free equilibrium*.

### Recurrent TB: Model with all reinfection pathways (*A*, *B* and *C*) *θ* > 0: Cubic *P*_1_(λ)

We now return to the fully general model with all parameters *p*, *σ*, *θ* positive. Recall that the sign of the roots of the cubic polynomial (Eq (4)) *P*_1_(λ), tell us the signs of the equilibrium populations for the number of infected individuals (via Eq (2)). Observe that the coefficients in the cubic polynomial *a*_3_, *a*_2_, *a*_1_ and *a*_0_ (see Eq (5)) are all real numbers. For any non-negative model parameters, *a*_3_ is always positive while *a*_2_, *a*_1_ and *a*_0_ can be either positive, zero or negative depending on *β*_0_, *β*_1_ and *β*_*R*_, respectively (see Eqs (6)–(8)). A comprehensive analysis of the roots of the cubic (9) may be carried out using Descartes rule of signs as described in S4 Appendix. A simpler more intuitive approach is to examine the roots at the transcritical bifurcation point *R*_0_ = 1. Such an analysis gives an understanding of the type of bifurcation that is likely to occur in the vicinity of *R*_0_ = 1, and provides conclusions that coincide with the more detailed analysis based on Descarte’s rule of signs (S4 Appendix). Conveniently when *R*_0_ = 1 the cubic polynomial (9) reduces to the quadratic equation

$$\begin{array}{c} f\left(\lambda \right)=a_{3}\lambda^{2}+a_{2}\lambda +a_{1}=0. \end{array}$$

**Fig 6:**
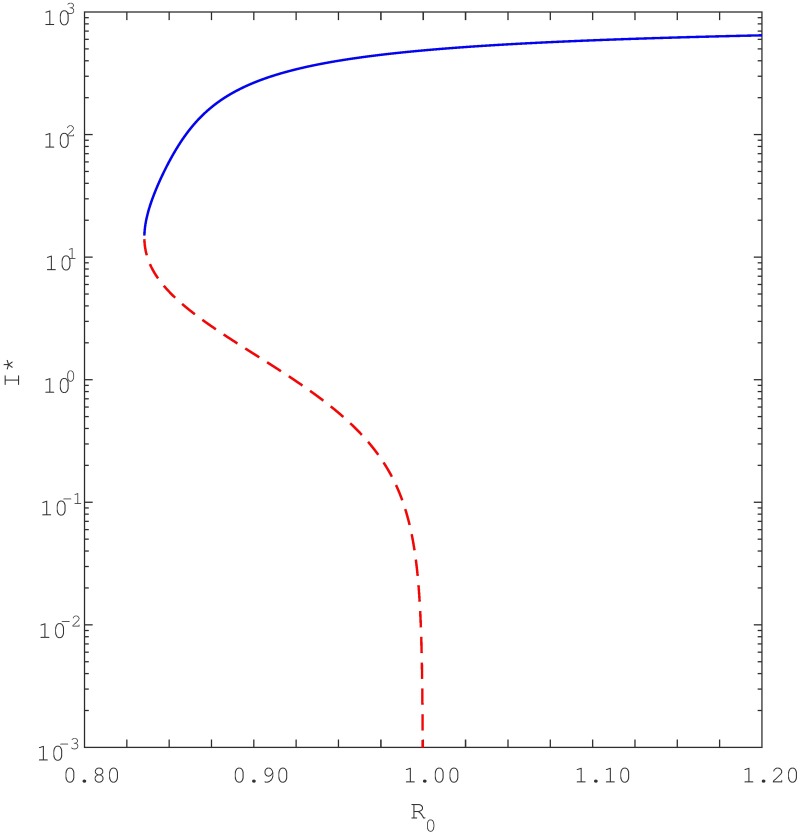
Infected population, I* at equilibrium plotted as a function of the parameter *β*. Illustrates backward bifurcation when recurrent TB parameters are included. Parameters used include; *p* = 0.09 > *p*_*c*_ = 0.0639., *σ* = 0.05, *θ* = 0.3, *μ* = 0.0167, *μ*_*d*_ = 0.1, *r* = 2, *q* = 0.05, Λ = 100 *c* = 60, *k* = 0.0002 and *β* ∈ {0.3, 0.8}. Note that the blue solid line denote stable equilibria while dotted red line denote unstable equilibria. To aid visualisation, bifurcation structure is plotted with semi-log axes.

**Fig 7:**
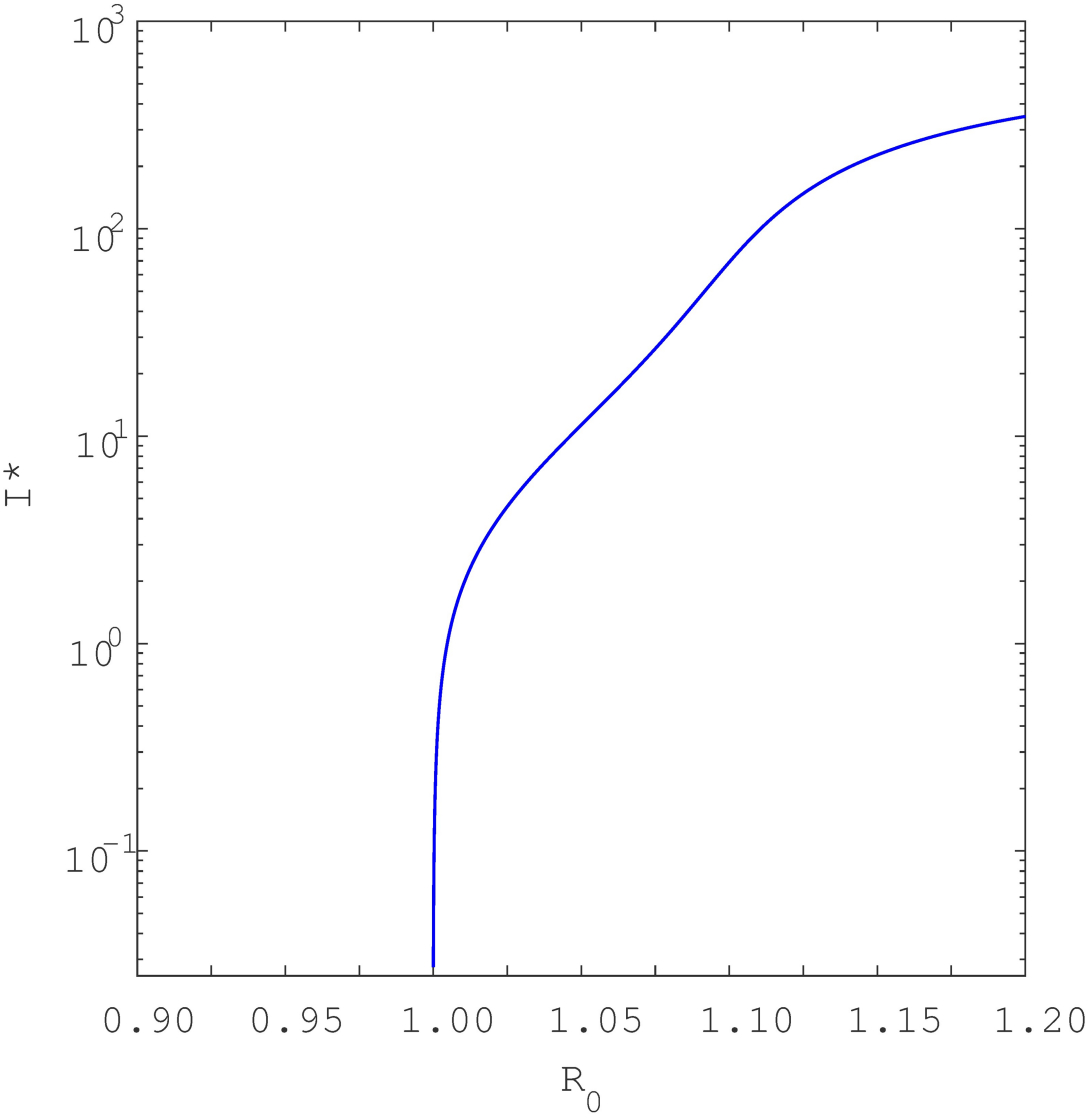
Infected population, I* at equilibrium plotted as a function of the parameter *β*. Shows forward bifurcation when recurrent TB parameters are included. Parameters used are same as in Fig 6 except *p* = 0.06 < *p*_*c*_ = 0.0639. To aid visualisation, bifurcation structure is plotted with semi-log axes.

**Fig 8:**
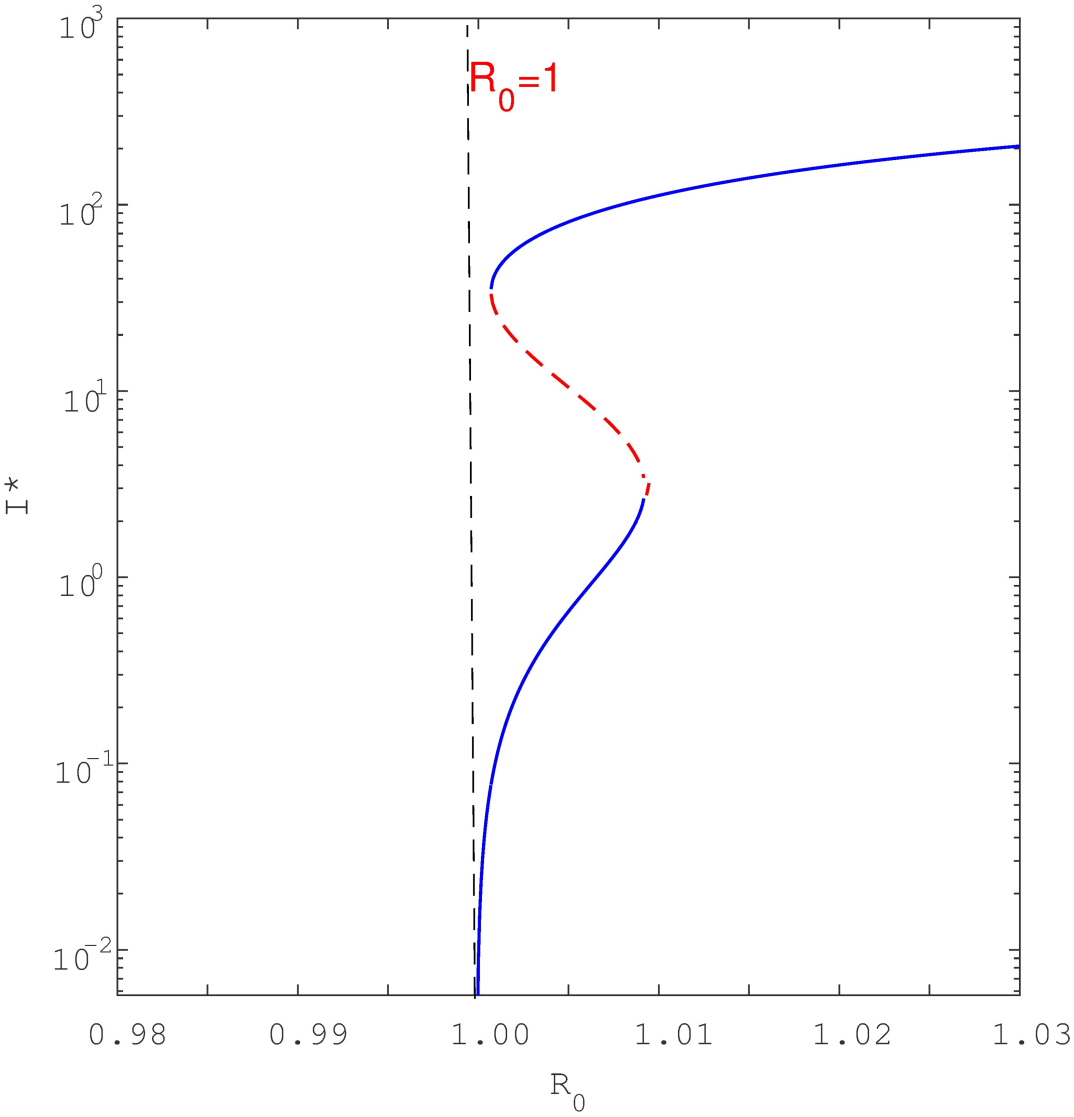
Shows forward bifurcation with hysteresis where the multiple equilibria are strictly to the right of *R*_0_ = 1. Parameters: *μ* = 0.0167, *μ*_*d*_ = 0.1, *k* = 0.0002, *θ* = 0.5, *r* = 2, *q* = 0.05, *σ* = 0.2, *c* = 60, Λ = 100, *β* ∈ (0.2, 0.8) and *p* = 0.057 < *p*_*c*_ = 0.0639. Note that the blue solid line denote stable equilibria while dotted red line denote unstable equilibria. To aid visualisation, the bifurcation structure is plotted with semi-log axes.

**Fig 9:**
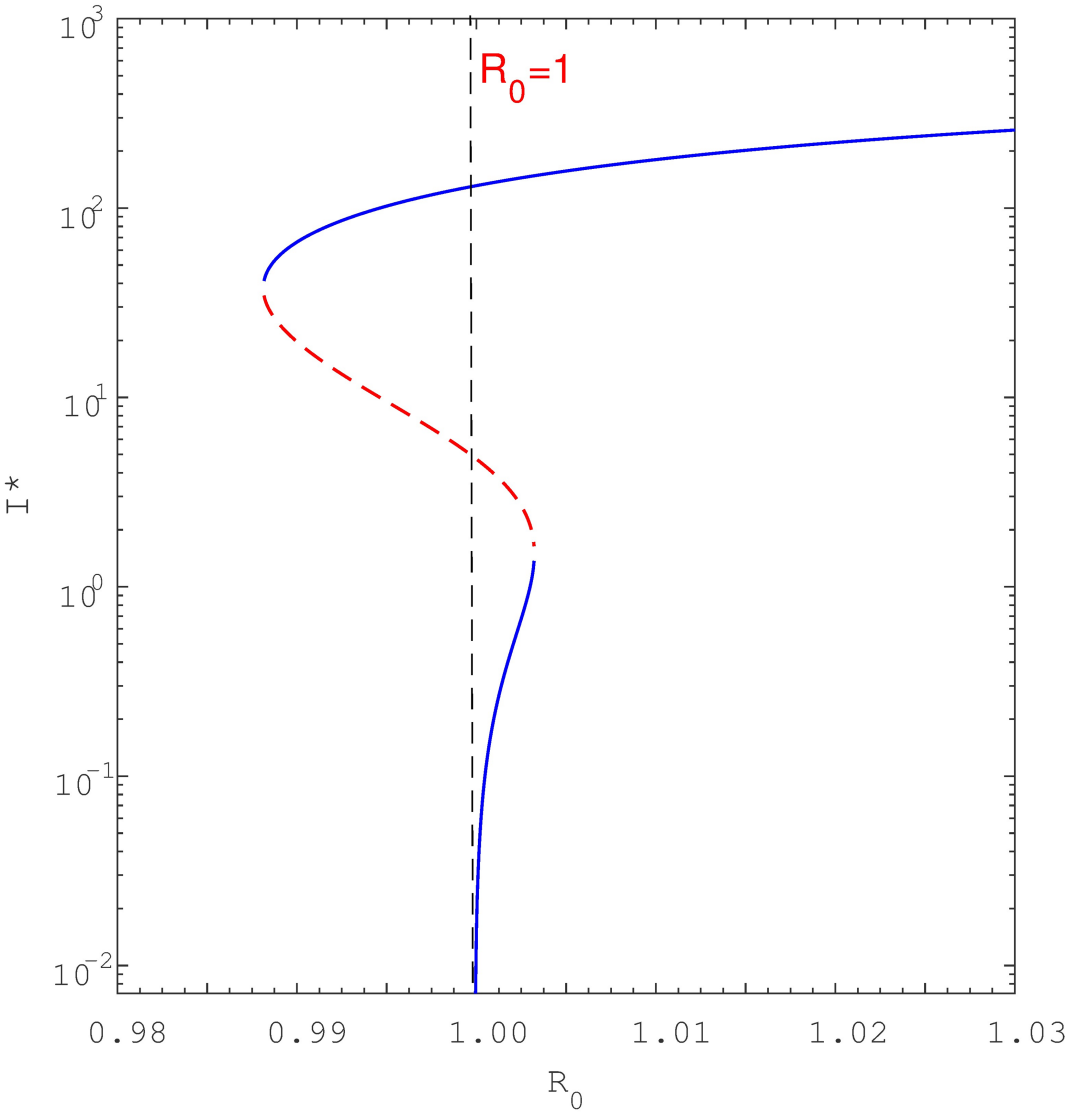
Shows forward bifurcation with hysteresis where there are multiple equilibria to the left and to the right of *R*_0_ = 1. Parameters used are similar to Fig 8 except *p* = 0.058 < *p*_*c*_ = 0.0639. For a clear view, the bifurcation structure is plotted with semi-log axes.

Keeping in mind Eq (5), a simple study of the roots shows that if either *a*_2_ < 0 and *a*_1_ < 0 (i.e., if *β* > *β*_0_ and *β* > *β*_1_) or if *β*_1_ < *β* < *β*_0_, the quadratic equation has one positive endemic equilibria. As seen in Fig 6, this is the signature of a backward bifurcation. Namely, when *R*_0_ is slightly below unity, the model Eq (1) has two positive endemic equilibria but only one when *R*_0_ ≥ 1. Furthermore, if *a*_2_ > 0 and *a*_1_ > 0 (i.e *β* < *β*_0_ and *β* < *β*_1_) then at the point *R*_0_ = 1, the model has no positive endemic equilibria. This characteristic is indicative of forward bifurcation as seen in Fig 7. However, if *R*_0_ is increased slightly above unity then model Eq (1) has one positive endemic equilibrium point. It can be shown that if *β*_0_ < *β* < *β*_1_, then the reduced equation has two positive real roots, indicating that the model Eq (1) exhibits hysteresis (see Figs 8 and 9), as will be discussed in detail shortly.

This discussion based on the point *R*_0_ = 1 is summarized in Lemma 2.

**Lemma 2** *At the point R*_0_ = 1, *where β* = *β*_*R*_ *the model* Eq (1) *has*:

*(i) Two positive endemic equilibria if β*_0_ < *β*_*R*_ < *β*_1_, *which signals hysteresis*,
*(ii) One positive endemic equilibria if β*_*R*_ > *β*_0_ *and β*_*R*_ > *β*_1_, *or if β*_1_ < *β*_*R*_ < *β*_0_, *either of which signals backward bifurcation*,
*(iii) No positive endemic equilibria if β*_*R*_ < *β*_0_ *and β*_*R*_ < *β*_1_, *which signals forward bifurcation*.

### Existence of backward bifurcation reinfection threshold at *R*_0_ = 1

The concept of existence of a critical point at *R*_0_ = 1 was fully developed by [49]. For the general model (1), we are interested in determining the critical value of exogenous reinfection *p*_*c*_ required to allow the formation of a backward bifurcation at *R*_0_ = 1.

Define

$$\begin{array}{cc} & p_{c}=(\frac{k+\mu q}{\mu (1-q)})(\frac{\mu \left(1-q\right)\left(\mu +r+\mu_{d}\right)+\left(\mu +r\right)\left(k+\mu q\right)}{\mu (\mu +r+\mu_{d})}-F_{r})\; \end{array}$$ (13)

where

$$\begin{array}{cc} & F_{r}=\frac{r\theta (k+\sigma \mu )}{\mu (\mu +r+\mu_{d})}. \end{array}$$ (14)

The expression *F_r_* (Eq 14) is associated with recurrent TB since it is the only term containing the reinfection parameter *θ*. In the absence of recurrent TB due to reinfection (i.e *θ* = 0) the expression *F_r_* reduces to zero. We now state the following Lemma 3:

**Lemma 3** *The model system* (1) *at R*_0_ = 1 *exhibits*,

*(i) Backward bifurcation whenever p* > *p*_*c*_,
*(ii) Forward bifurcation whenever p* < *p*_*c*_.

To see this, first note that referring to Fig 6, it is apparent that for our model a backward bifurcation requires that at *R*_0_ = 1 there is a single positive endemic equilibrium. Note again that when *R*_0_ = 1 (where *a*_0_ = 0), the equilibria relate to the roots of the quadratic *f*(λ) = *a*_3_ λ^2^ + *a*_2_ λ + *a*_1_ = 0. The roots are:

$$\begin{array}{c} \lambda =\left(-a_{2}\pm \sqrt{a_{2}^{2}-4a_{1}a_{3}}\right)/2a_{3}\;\text{where}\;\;a_{3}>0. \end{array}$$ (15)

We are interested in the point where the bifurcation changes from forward to backward. But when *R*_0_ = 1 (*β* = *β*_*R*_) this is just the point where the positive endemic equilibrium vanishes to zero, and from Eq (15) must occur when *a*_1_ = 0 (*β* = *β*_1_); or equivalently *β*_1_ = *β*_*R*_ (see Eq (5)). Also note that *β*_1_ is a function of *p* (i.e. *β*_1_ = *β*_1_(*p*)) while *β*_*R*_ is not. We therefore equate Eqs (7) and (8) for *β*_1_(*p*) and *β*_*R*_ and solve to find the critical value *p*_*c*_ for which *β*_1_(*p*_*c*_) = *β*_*R*_. After some algebraic manipulation we find the required backward bifurcation reinfection threshold is given by Eq 13. A more detailed derivation of *p*_*c*_ can be found in S2 Appendix, which takes into account the stability of the equilibria through the use of center manifold theory.

Numerically, Lemma 3 is illustrated by Figs 6 and 7 which respectively show that model Eq 1 has a backward bifurcation when *p* > *p*_*c*_ and forward bifurcation when *p* < *p*_*c*_.

### Relation with models of Lipsitch & Murray [18] and Feng et al. [23]

It is interesting to compare the critical point *p*_*c*_ for the reinfection threshold given in Eq (13) with that found by Feng et al. [23] in their much simpler but still important model. First note that Feng et al. [23] do not include fast primary progression which is equivalent to setting *q* = 0 (see Fig 3). They also assume that the exogenous reinfection rates are *θ* = 1, *σ* = 0 and that the disease induced death rate is negligible with *μ*_*d*_ = 0. Under these conditions Feng et al. [23] find that backward bifurcations occur only if *p* > *P*_*feng*_, where

$$\begin{array}{c} P_{feng}=\frac{k}{\mu }(1+\frac{k}{\mu +r})\approx \frac{k}{\mu }, \end{array}$$

and the latter approximation assumes that *k* ≪ *r*.

In their controversial paper, Lipsitch and Murray [18] argued that in the real world recovered individuals gain immunity to reinfection, and thus reinfection amongst exposed individuals must be less than the probability of progressing to the infectious stage of TB. They showed that this implies

$$\begin{array}{c} p<P_{L}=k/\left(\mu +k\right)\approx \frac{k}{\mu }. \end{array}$$

given that *k* ≪ *μ*. As such, Lipsitch and Murray [18] argued that backward bifurcations should not be expected in the real world.

Our extended model has some interesting insights with regard to these studies. First note that after inclusion of the assumptions made by Feng et al. [23] in our model (eg., setting *q* = 0), the threshold *p*_*c*_ for backward bifurcation (Eq (13)) simplifies to

$$\begin{array}{c} p_{c}\approx P_{feng}. \end{array}$$ (16)

That is, backward bifurcations are possible only when *p* > *p_c_* ≈ *p_feng_*, and thus the result of Feng et al. [23] is retrieved.

Consider now the extended model with more realistic reinfection pathways (0 < *q* ≪ 1, *σ* > 0), but still assuming that *μ*_*d*_ = 0, *k* ≪ *r* and *μ* ≪ *r*. For this approximation *F*_*r*_ ≈ *σθ*, and the backward bifurcation threshold Eq 13 simplifies to:

$$\begin{array}{c} p_{c}\approx (q+\frac{k}{\mu })(1+\frac{k}{\mu }-\sigma \theta )\\ \approx (\frac{k}{\mu }+q)\left(1-\sigma \theta \right). \end{array}$$ (17)

It is interesting to compare this result to the Lipsitch and Murray [18] threshold *P*_*L*_ discussed above. If recovered individuals gain high immunity from having been infected, then the reinfection pathway *R* → *I* is relatively small (see Fig 3), with *σθ* ≪ 1. In this case Eq (17) shows that *p*_*c*_ ≈ *P*_*L*_ + *q*. This is similar to the [18] criterion, and suggests that backward bifurcation is unlikely to occur in the real world, if as Lipsitch and Murray [18] claim that in reality *p* < *P*_*L*_. However, if recovered individuals gain only mild immunity against reinfection, then the reinfection pathway *R* → *I* and *σθ* can be relatively large. Note that *θ* can be greater than unity as suggested by Verver et al. [13]. Gomes et al. [41] estimate *θ* to be in the range [1.61, 7.79]. In this situation it is quite possible that *p*_*c*_ < *P*_*L*_. Hence, with relatively low immunity amongst recovered individuals [13] backward bifurcation can occur despite the fact that the Lipsitch and Murray [18] prediction would literally predict otherwise. This does not mean that Lipsitch and Murray [18] have erred, but that their result may need modifications when discussing the presence of more complex reinfection pathways. In fact even just for the simplified reinfection pathways of the original Feng et al. [23] model, the validity of the Lipsitch and Murray [18] argument has recently been called into question given the difficulties of comparisons with real world processes (Wang et al. [32]).

In the more recent literature, numerous studies have pointed out that initial infection may not confer protection against exogenous reinfection especially in high-risk populations [50, 51]. Some studies have demonstrated that it is possible for exogenous reinfection to outweigh endogenous reinfection [20, 21]. This is supported by the fact that the majority of new TB cases (about 90%) occur as a result of reinfection rather than endogenous reactivation [19–21, 43]. In this situation, *p*_*c*_ > *k*/(*μ* + *k*), and backward bifurcations can occur even according to the Lipsitch and Murray [18] criteria. Other medical research shows that reinfection rates after successful treatment are much higher than rates of new TB; sometimes approximately four times higher [13, 41]. Furthermore, similar to vaccine conferred immunity, the protection rendered by latent TB infection wanes with time and it is uncertain whether latent infections would provide a similar immunity decades after the first episode of TB [43].

## Hysteresis

For the usual forward bifurcation (eg., Fig 4), a model has two locally stable branches at the transcritical point *R*_0_ = 1: *i)* an infection free equilibrium that is locally asymptotically stable when *R*_0_ < 1 and *ii)* an endemic equilibrium which is stable for *R*_0_ > 1. However, the scenario where there is only one endemic equilibria when *R*_0_ > 1 may not always be the case. For example, Reluga et al. [52] noted in their study of epidemic models with structured immunity that it is possible that more than one endemic equilibria may coexist even though the basic reproduction number *R*_0_ > 1. This leads to an unusual phenomenon of forward bifurcation with hysteresis, which can be triggered in our TB model when reinfection is taken into account. Thus Eq (1) exhibits a hysteresis effect where multiple endemic equilibria coexist when *R*_0_ > 1, as shown in Fig 8. The two ‘outer’ equilibria are stable while the interior equilibrium (red) is unstable. Table 2 clarifies that three endemic equilibria coexist for *R*_0_ > 1 if *β*_0_ < *β* < *β*_1_. For some parameter regimes with hysteresis, the endemic equilibria may also be found in the region where *R*_0_ < 1 and where disease is not expected, as shown in Fig 9. In this scenario, (similar to a backward bifurcation) TB persists for *R*_0_ < 1 even though the bifurcation at *R*_0_ = 1 is a forward bifurcation. We know of no other epidemic modelling study reporting this feature. Hysteresis loops to the left of the epidemic threshold *R*_0_ = 1 have epidemiological implication in that, although there is no backward bifurcation (as ascertained by the fact that the hysteresis effect occurs when (*p* < *p*_*c*_)) policy makers need to reduce *R*_0_ below another threshold *R*_*c*_ to eradicate TB. That is, reducing *R*_0_ below unity will be necessary but not sufficient in eradicating TB within the community.

**Table 2:** Generalization of the model equilibria of Eq (1).

Range of *R*_0_	Conditions	Equilibria of model system (1)	Type of bifurcation
*R*_0_ = 1	*β* > *β*_0_, *β* > *β*_1_	One positive endemic equilibrium	Forward bifurcation
	*β*_1_ < *β* < *β*_0_	One positive endemic equilibrium	Forward bifurcation
	*β*_0_ < *β* < *β*_1_	Two positive endemic equilibria	Associated to hysteresis
*R*_0_ > 1	*β* ≥ *β*_0_, *β* ≥ *β*_1_	One positive endemic equilibrium	Forward bifurcation
	*β* ≤ *β*_0_, *β* ≤ *β*_1_	One positive endemic equilibrium	Forward bifurcation
	*β*_0_ < *β* < *β*_1_	Three positive endemic equilibria	Hysteresis
*R*_0_ < 1	*β* > *β*_0_, *β* > *β*_1_	Two positive endemic equilibria	Backward bifurcation
	*β*_1_ ≤ *β* < *β*_0_	Two positive endemic equilibria	Backward bifurcation
	*β* < *β*_0_, *β* < *β*_1_	No positive endemic equilibria	Associated to forward bifurcation

## No reinfection path *A*, (i.e *p* = 0, *θ* > 0)

The case when there is no reinfection among recovered individuals but reinfection of exposed individuals (i.e *p* > 0 and *θ* = 0) was studied by Kar et al. [22], where an exogenous reinfection threshold was established. In this section we focus on the scenario when there is no exogenous reinfection among exposed individuals (i.e *p* = 0, reinfection path A is omitted). Our goal is to demonstrate that in such a situation recurrent TB due to reinfection of recovered individuals only (*θ* > 0) can induce the phenomenon of backward bifurcation.

In model system (1) setting *p* = 0 we obtain the following subsystem:

$$\begin{array}{ccc} \frac{dS}{dt} & = & \Lambda -\lambda S-\mu S,\\ \frac{dE}{dt} & = & (1-q)\lambda S+(1-\sigma )\theta \lambda R-(\mu +k)E,\\ \frac{dI}{dt} & = & q\lambda S+\sigma \theta \lambda R+kE-(\mu +r+\mu_{d})I,\\ \frac{dR}{dt} & = & rI-\theta \lambda R-\mu R. \end{array}$$ (18)

The model system (18) equilibrium points (S*, E*, I*, R*) can be expressed in terms of force of infection λ*, obtained by solving the following quadratic equation;

$$\begin{array}{c} P_{3}\left(\lambda^{*}\right)=b_{2}\lambda^{*2}+b_{1}\lambda^{*}+b_{0}=0, \end{array}$$ (19)

$$\begin{array}{c} b_{2}=\left(1-q\right)\left(\mu +\mu_{d}\right)\theta +\left(1-\sigma \right)\theta r+\theta \left(k+\mu q\right),\\ b_{1}=\theta \left(\mu +\mu_{d}\right)\left(\mu +k\right)+r\theta \mu \left(1-\sigma \right)+\left(1-q\right)\left(\mu +\mu_{d}\right)\mu +r\left(\mu +k\right)+\mu \left(k+\mu q\right)-c\beta \left(q\theta \mu +\theta k\right),\\ b_{0}=\mu \left(\mu +r+\mu_{d}\right)\left(\mu +k\right)\left(1-R_{0}\right). \end{array}$$

Note that subsystem (18) has the same basic reproduction number as model (1).

It is easy to see that *R*_0_ = 1 implies *b*_0_ = 0. Thus, the following equality is satisfied;

$$\begin{array}{c} \left(\mu +r+\mu_{d}\right)\left(\mu +k\right)=\beta c\left(k+\mu q\right). \end{array}$$ (20)

This combined with the condition *b*_1_ < 0, which is necessary for backward bifurcation to occur, and with some algebraic manipulation leads to the criterion for backward bifurcation:

$$\theta >\frac{\left(1-\sigma )(\mu +\mu_{d})\mu +r(\mu +k)+\mu (k+\mu q\right)}{r(k+\mu \sigma )}≜\theta_{c}.$$ (21)

Thus, we arrive at the following Theorem;

**Theorem 2** *The model subsystem* (18) *at R*_0_ = 1 *has*:

*(i) Backward bifurcation if θ* > *θ_c_*
*(ii) Forward bifurcation if θ* < *θ_c_*

**Fig 10:**
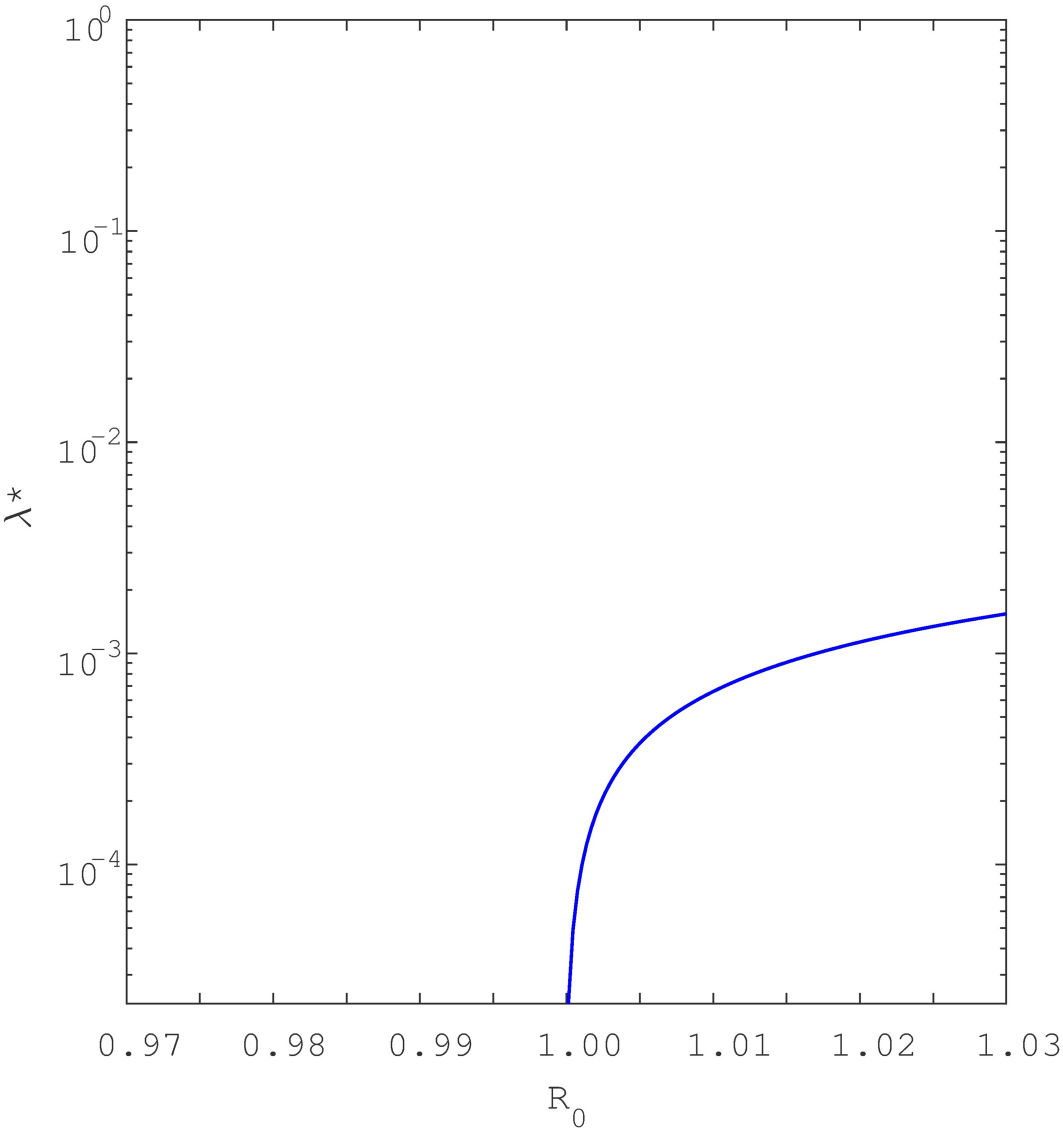
Shows forward bifurcation in a scenario where reinfection path *A* is omitted (i.e no reinfection of exposed individuals, *p* = 0). Parameters used include: *μ* = 0.0167, *μ*_*d*_ = 0.1, *k* = 0.0002, *r* = 2, *q* = 0.05, *σ* = 0.2, *c* = 60, *β* ∈ (0.5, 0.65) and *θ* = 4 < *θ*_*c*_ = 5. Semi-logarithmic scales are used to aid visualisation.

Furthermore, a similar condition to (21) can be obtained by setting *p* = 0 in the center manifold results of S2 Appendix, hence corroborating Theorem 2. Thus, if *θ* > *θ*_*c*_ = 5 model (18) will exhibit backward bifurcation. However, if *θ* < *θ*_*c*_ = 5 model (18) does not exhibit backward bifurcation (i.e has only forward bifurcation) for the parameters given in Fig 10 caption.

With the existing evidence that recovered individuals have increased susceptibility to reinfection, four times higher than that of new TB [13, 27], Theorem 2 suggests that the contribution of recurrent TB in the general TB burden can significantly alter TB dynamics especially in a scenario where recurrent TB independently triggers the phenomenon of backward bifurcation.

**Fig 11:**
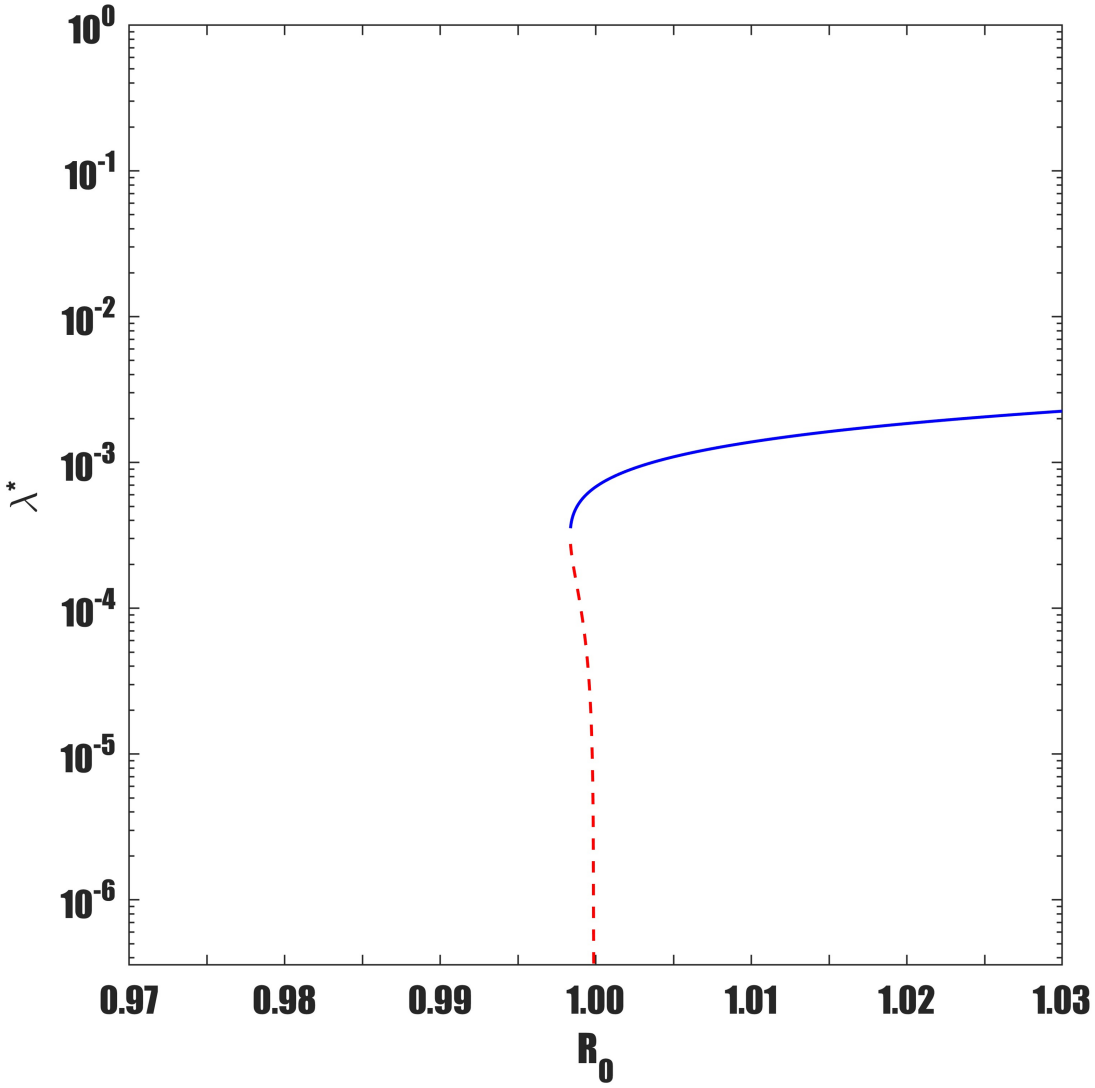
Shows backward bifurcation structure when reinfection path *A* is excluded. Parameters used are the same as in Fig 10 except *θ* = 6 > *θ*_*c*_ = 5. The solid blue line represent stable equilibrium while the dotted red line represent unstable equilibrium. Semi-logarithmic scales are used to aid visualisation.

**Fig 12:**
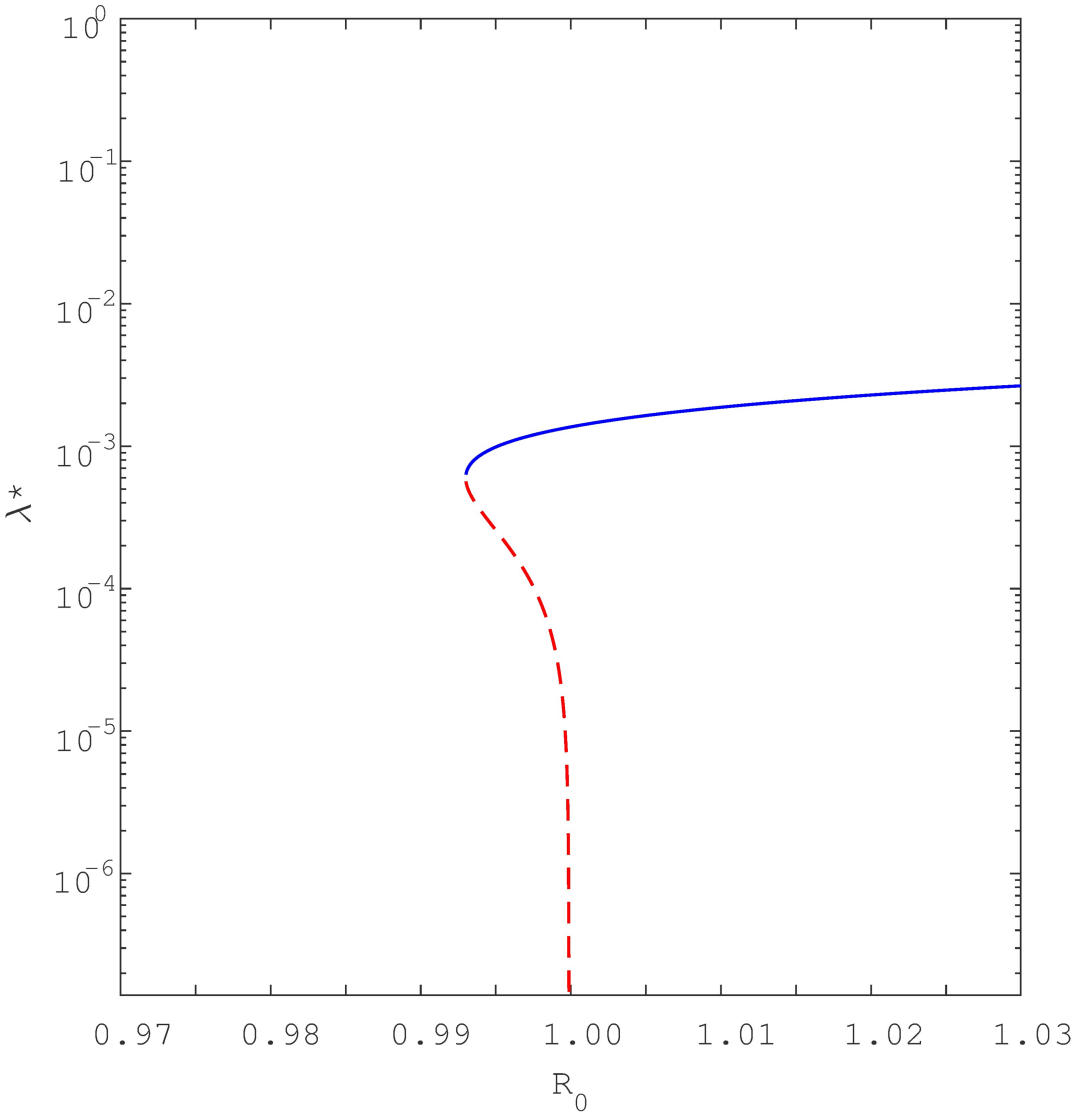
Shows backward bifurcation structure when reinfection path *A* is excluded. Parameters used are the same as in Fig 10 except *θ* = 7.5 > *θ*_*c*_ = 5. The solid blue line represent stable equilibrium while the dotted red line represent unstable equilibrium. Semi-logarithmic scales are used to aid visualisation.

It is important to note that although previous TB models have attempted to incorporate recurrent TB pathways they do not investigate whether recurrent TB alone can trigger bi-stability, but rather concentrate on backward bifurcation caused by exogenous reinfection of exposed individuals (i.e *p* > 0). Selecting value of parameter *θ* from the estimated interval, i.e *θ* ∈ 3.87[1.61, 7.79] [41], we verify the threshold given in Theorem 2. Fig 10 is a bifurcation diagram corresponding to the case *θ* < *θ*_*c*_ and indicates a forward bifurcation, as predicted by Theorem 2 Case (ii). Similarly, Figs 11 and 12 indicate backward bifurcation since *θ* > *θ*_*c*_ as predicted by Case (i).

### Impact of incorporating recurrent TB parameters

**Fig 13:**
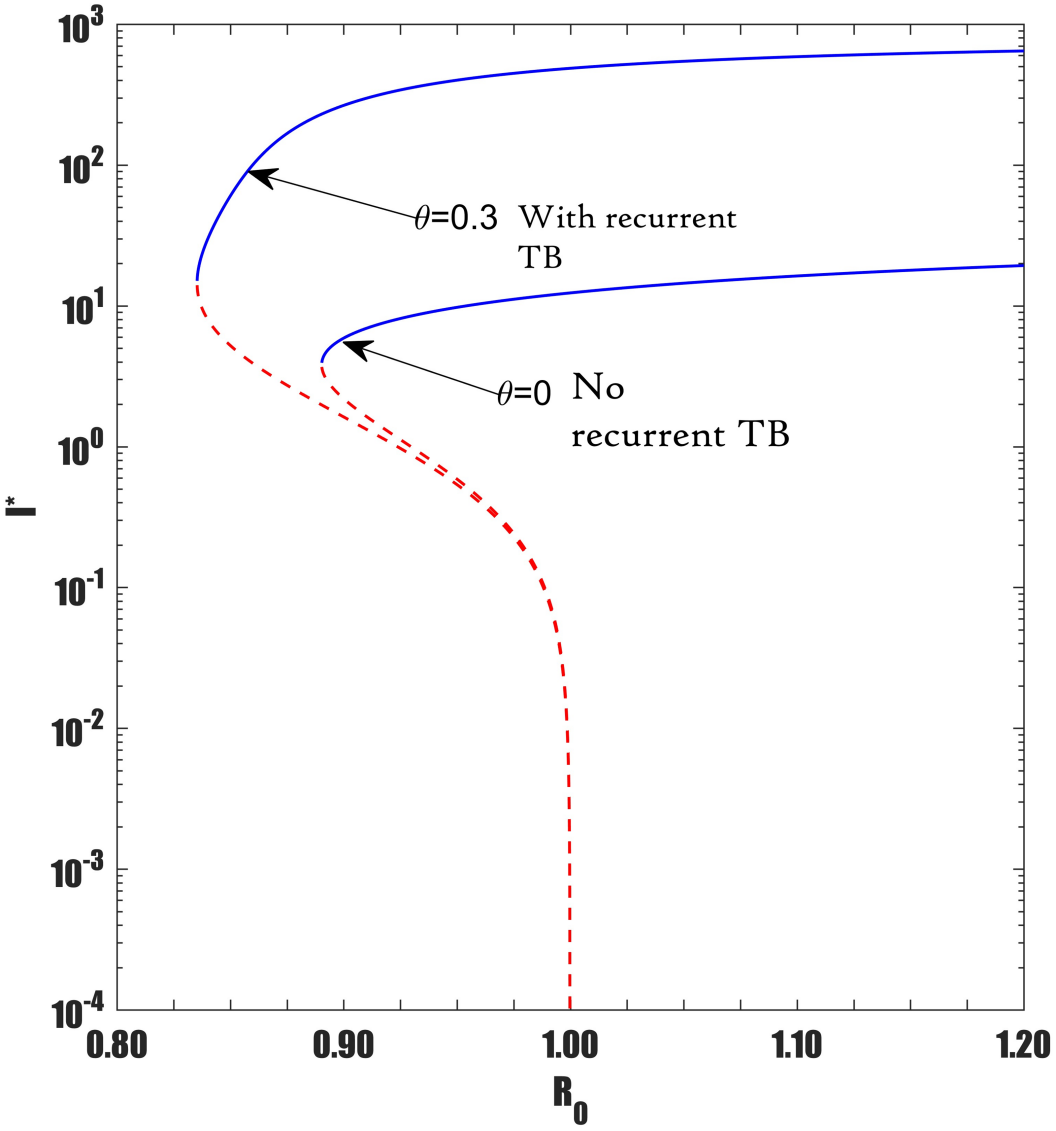
Shows the effect of recurrent TB on backward bifurcation due to incorporation of reinfection pathways B and C. Parameter values: *μ* = 0.0167, *μ*_*d*_ = 0.1, *k* = 0.0002, *r* = 2, *q* = 0.05, *σ* = 0.05, *p* = 0.09, *c* = 60, Λ = 100, *β* ∈ [0.45, 0.7]. With no recurrent TB (*θ* = 0), *p* = 0.09 > *p*_*c*_ = 0.0658 while with recurrent TB (*θ* = 0.3). Parameters used are the same but *p*_*c*_ is altered, i.e *p* = 0.09 > *p*_*c*_ = 0.0647. The blue solid line represent stable equilibria while the dotted red line represent unstable equilibria. Semi-log scales are used for a clear view.

**Fig 14:**
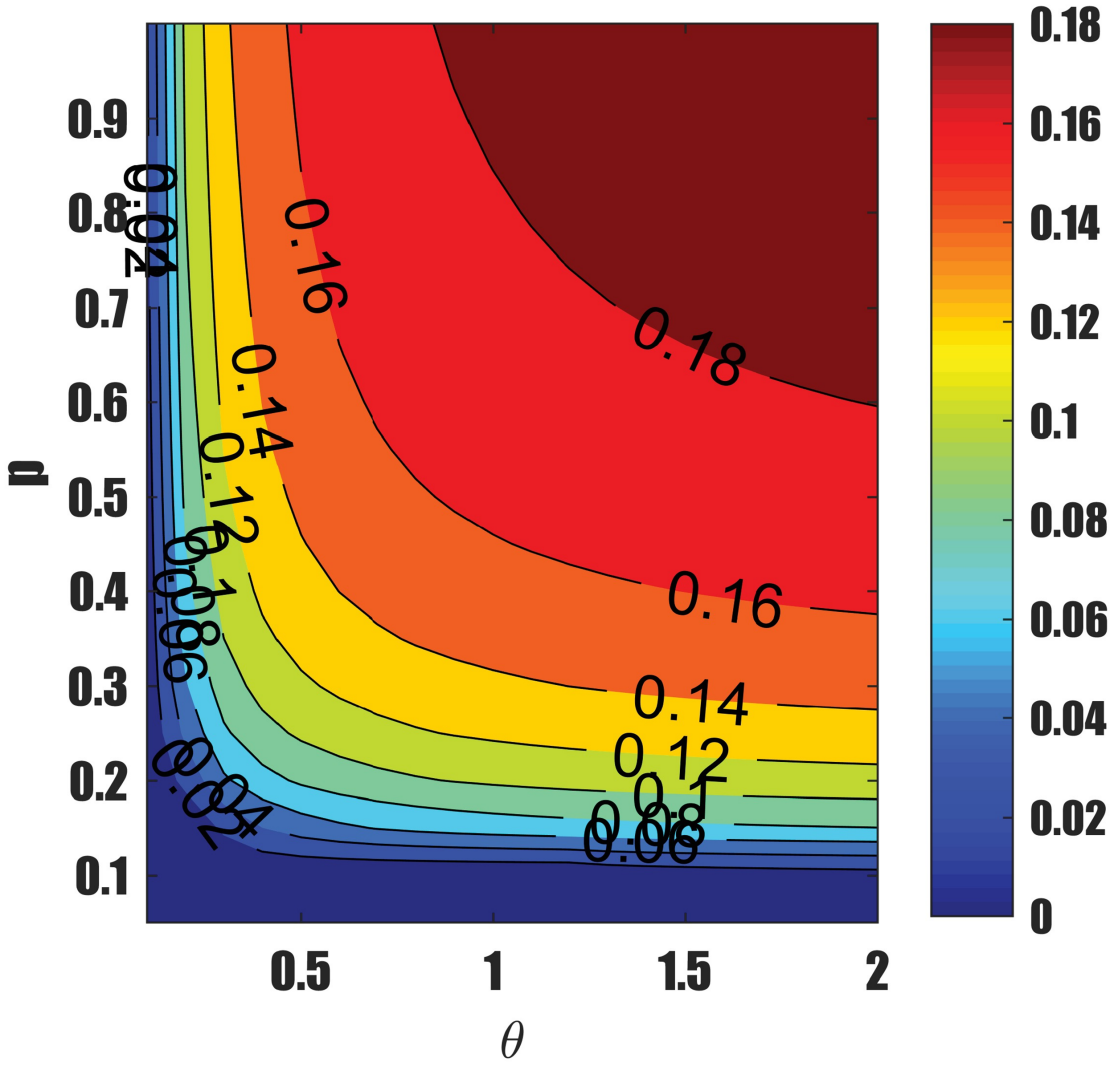
Contour plots of force of infection at equilibrium as a function of *θ* and *p*. Increasing recurrent TB increases TB prevalence.

Recall that recurrent TB due to reinfection is denoted by reinfection pathways *B* and *C* in Fig 3. Given that reinfection path *A* does induce backward bifurcation when *p* > *p*_*c*_ it is of interest to investigate how the additional recurrent reinfection paths *B* and *C* can impact the backward bifurcation. In Fig 13 the endemic equilibrium I* is plotted as a function of the basic reproduction number *R*_0_ for scenarios with different recurrent TB contributions. The figure shows that recurrent TB due to reinfection among treated/recovered individuals shifts the backward bifurcation to the left as well as widens the gap between bifurcation curves. In summary we report the following important implications that arise from our analysis. Raising the intensity of recurrent TB:

(i) Widens the gap between the bifurcation curves,
(ii) Reduces the critical value *R*_*c*_,
(iii) Increases the number of infected individuals (TB burden) (see Fig 14),
(iv) Reduces the reinfection threshold *p*_*c*_ that induces the phenomenon of backward bifurcation.

## Discussion

Until just before the turn of the century the role of exogenous reinfection in the transmission of TB was usually believed to be minimal. van Rie et al. [50] wrote: “For decades it has been assumed that postprimary tuberculosis is usually caused by reactivation of endogenous infection rather than by a new, exogenous infection.” However, these views are no longer considered accurate and our understanding of the role of exogenous reinfection has been completely revised. Warren et al. [53] refuted the unitary concept of pathogenesis of tuberculosis proposed in 1960s: that is, tuberculosis results from a single infection with a single *Mycobacterium tuberculosis* strain, and such infections were thought to confer protective immunity against exogenous reinfection. Thus, exogenous reinfection was thought to be uncommon. Murray et al. [19] found that their data for exogenous reinfection among US immigrants strongly suggested that reinfection likely plays a major role in high-incidence TB areas.

Lipsitch and Murray [18] argued that backward bifurcation should not occur when taking into account biologically realistic parameter values. Their argument was built on the premise that exogenous reinfection among exposed individuals should be less than the probability of progressing to clinically active TB due to endogenous reactivation, which translates in mathematics to *p* < *P*_*L*_ = *k*/(*μ* + *k*). But Feng et al. [23] found that backward bifurcation can only take place if *p* > *P*_*feng*_ > *P*_*L*_. For this reason, Lipsitch and Murray [18] concluded that backward bifurcations are unlikely to be relevant in the context of TB despite non-existence of a data to support their claim. The argument of Lipsitch and Murray [18] was based on the Feng et al. [23] TB model which as mentioned, failed to incorporate key TB pathways such as primary progression and recurrent TB due to reinfection where some recovered/treated individuals revert directly to the infective stage. Yet, these pathways are critical to TB epidemiology, an aspect that was even pointed out by Lipsitch and Murray [18] as a weakness of the Feng et al. [23] model. Lipsitch and Murray [18] called for further research that would account for these omitted pathways. However, modern research no longer seems to support Lipsitch and Murray’s [18] argument since exogenous reinfection among individuals with latent TB can outweigh endogenous reactivation [20, 21]. And this implies that the protection provided by latent TB infection is not strong enough to prevent individuals becoming reinfected. This is supported by the fact that majority of new TB cases (about ninety percent) are as a result of reinfection rather than endogenous reactivation [13, 19–21]. Moreover, studies of TB reinfection in high-HIV burden countries have demonstrated the strong negative impact of HIV on immunity, which is likely to outweigh the possible protection conferred by latent infection [43]. Backward bifurcation is likely to occur in this scenario, since reinfection will be greater than endogenous reactivation (i.e, *p* > *k*/(*μ* + *k*)).

The analysis conducted here has shown that when exogenous reinfection is significant (resulting in relatively small *p*_*c*_—see S5 Appendix), as is currently understood to be not atypical, backward bifurcation can indeed occur for relatively low values of the reinfection parameter *p*. Furthermore, we observed that if we omit the exogenous reinfection path *A* required to cause backward bifurcation in the study of Feng eta al. [23], then recurrent TB alone (i.e., paths *B* and *C* in our model) may still yield backward bifurcations. For instance, if *p* = 0 (thereby omitting pathway A), and the recurrent TB rate parameter *θ* exceeds a certain threshold *θ* > *θ*_*c*_ then backward bifurcation can indeed occur (see Figs 11 and 12). To the best of our knowledge, this result has not been observed in previous TB modelling studies. In addition, recurrent TB can induce forward bifurcation with hysteresis, rather than just the usual forward bifurcation. Interestingly, the hysteresis loop depends on reinfection parameters, and this may lead to an unusual scenario where the hysteresis crosses the threshold *R*_0_ = 1 thus entering the region where only backward bifurcation is expected (see Fig 9). Epidemiologically this implies that TB will persist when *R*_0_ < 1 even though we have only a forward bifurcation.

In the literature it has been observed that individuals who previously have had active TB and who were successfully treated are more likely to gain active TB another time [13]. However there is little understanding as to why this occurs. Gomes et al. [41] suggested two alternative mechanisms that might explain this: a) previous infection increases susceptibility of individuals to reinfection; b) population heterogeneity, in which some individuals are more at-risk than others, might lead to this conclusion. In this paper, we have almost exclusively explored the former possibility. However the latter possibility may also be at play as examined by Gomes et al. [41]. In the latter case, heterogeneity would be less likely to create changes in reinfection parameters such as *θ*, and thus might not lead to the same bifurcation phenomena found in this study. Nevertheless, a wealth of theoretical studies do suggest that heterogeneous infection processes are often involved in creating backward bifurcations, and thus we similarly expect complex dynamical phenomena [54–56].

In future work, it would be interesting to additionally consider the possibility that some infected individuals who are treated do not become completely cured. This situation would lead to another cohort of individuals characterised by the fact that treatment has failed, and thus require adding an extra compartment which distinguishes complete recovery and incomplete recovery (of individuals who are infectious). The extra infectives from the latter compartment will tend to increase TB prevalence and thus widen the bifurcation curves. While this possibility is of importance, it falls outside the main scope of the present article, and would lead to a model that is very difficult to analyse mathematically.

## Supporting information

S1 Appendix Computation of basic reproduction number. (PDF)

S2 Appendix Detailed derivation of *p*_*c*_ in Lemma 3. (PDF)

S3 Appendix Equilibrium quantities expressed in terms of λ. (PDF)

S4 Appendix Descartes rule of signs. (PDF)

S5 Appendix Further discussion on parameters. (PDF)
