# Supplementary material for: Backward bifurcation and hysteresis in models of recurrent tuberculosis
Source: PLoS One. 2018 Mar 22;13(3):e0194256. doi: 10.1371/journal.pone.0194256 (PMC5863985; doi:10.1371/journal.pone.0194256)
Supplement: S1 Appendix — (PDF) [file pone.0194256.s001.pdf]

S1 Appendix. Computation of basic reproduction number. Using the notation in [1] we define $K = -T\Sigma^{-1}$ where K is the dominant eigenvalue, T is the transmission matrix representing entries of new infections and Σ is the transition matrix representing change in state (i.e., removal by death or due to recovery).

$$T = \begin{pmatrix} 0 & (1-q)\beta c \\ \\ 0 & q\beta c \end{pmatrix}, \quad \Sigma = \begin{pmatrix} -(k+\mu) & 0 \\ \\ k & -(\mu + r + \mu_d) \end{pmatrix}.$$

Thus,

$$K = \begin{pmatrix} \frac{k(1-q)\beta c}{(k+\mu)(\mu+r+\mu_d)} & \frac{(1-q)\beta c}{(\mu+r+\mu_d)} \\ \\ \frac{kq\beta c}{(k+\mu)(\mu+r+\mu_d)} & \frac{q\beta c}{(\mu+r+\mu_d)} \end{pmatrix}.$$

Now the associated basic reproduction number is defined as $R_0 = \rho(K)$ where ρ is the spectral radius is given as

$$R_0 = \frac{\beta c(k + q\mu)}{(\mu + k)(\mu + r + \mu_d)}.$$

Theorem 1 *The model (1) when $\sigma = \theta = 0$ has:*

(i) a unique positive endemic equilibria if $c_1 < 0$ and $R_0 = 1$, or if the discriminant $\triangle = c_1^2 - 4c_2c_0 = 0$;

(ii) a unique positive endemic equilibria if $R_0 > 1$;

(iii) two positive endemic equilibria if the three conditions hold: $c_1 < 0$, $R_0 < 1$ and $\triangle = c_1^2 - 4c_2c_0 > 0$, and thus backward bifurcation;

(iv) no positive endemic equilibria if $c_1 > 0$ and $R_0 \leq 1$

Proof of Theorem 1

It is easy to note that in polynomial (10) c_2 is always positive. Also $c_0 > 0$ if $R_0 < 1$. For Case (i) where $c_1 < 0$ and $R_0 = 1$ (i.e $c_0 = 0$) the quadratic Eq (10) in the text reduces to $P_2(\lambda) = c_2\lambda + c_1 = 0$ and in this case the model Eq (1) will have a unique positive endemic equilibrium, since $c_1 < 0$. (Note there would be no positive equilibrium if $c_1 \geq 0$.) Moreover, it is instructive to note that in Case (i), if the discriminant $\triangle = 0$, then the quadratic Eq (10) in the text has the repeated root $\lambda = \frac{-c_1}{2c_2}$. In such a case model Eq (1) has a unique positive endemic equilibrium if $c_1 < 0$, no positive endemic if $c_1 > 0$ and DFE if $c_1 = 0$ (i.e $\lambda = 0$ which corresponds to DFE). Similarly, the remaining cases in the proof follow directly from the trivial properties of the roots of quadratic polynomials.

References

[1] Diekmann O, Heesterbeek JAP, Roberts MG. The construction of next-generation matrices for compartmental epidemic models. Journal of the Royal Society Interface. 2009;p. rsif20090386.
