# Supplementary material for: Backward bifurcation and hysteresis in models of recurrent tuberculosis
Source: PLoS One. 2018 Mar 22;13(3):e0194256. doi: 10.1371/journal.pone.0194256 (PMC5863985; doi:10.1371/journal.pone.0194256)
Supplement: S2 Appendix — (PDF) [file pone.0194256.s002.pdf]

S2 Appendix Detailed derivation of p_c in Lemma 3. Proof. The proof employs center manifold approach as exhibited in center manifold theorem from Castillo-Chavez and Song [1]. For simplification and understanding of the center manifold theorem it is convenient to transform the model variables of system (1) as follows: $x_1 = S, x_2 = E, x_3 = I, x_4 = R$ and $N = \sum_{j=1}^{4} x_j$. Now letting $X = (x_1, x_2, x_3, x_4)^T$ (T denote transpose) the model system (1) can be written as $\frac{dX}{dt} = F(X)$ where $F = (f_1, f_2, f_3, f_4)^T$. Hence we have

$$\begin{aligned}
\frac{dx_1}{dt} &= \Lambda - \frac{\beta c x_1 x_3}{x_1 + x_2 + x_3 + x_4} - \mu x_1 = f_1,\\
\frac{dx_2}{dt} &= \frac{(1-q)\beta c x_1 x_3}{x_1 + x_2 + x_3 + x_4} + \frac{(1-\sigma)\theta\beta c x_3 x_4}{x_1 + x_2 + x_3 + x_4} - \frac{p\beta c x_2 x_3}{x_1 + x_2 + x_3 + x_4} - (\mu + k)x_2 = f_2, \qquad (1)\\
\frac{dx_3}{dt} &= \frac{q\beta c x_1 x_3}{x_1 + x_2 + x_3 + x_4} + \frac{\sigma\theta\beta c x_3 x_4}{x_1 + x_2 + x_3 + x_4} + \frac{p\beta x_2 x_3}{x_1 + x_2 + x_3 + x_4} + kx_2 - (\mu + r + \mu_d)x_3 = f_3,\\
\frac{dx_4}{dt} &= rx_3 - \frac{\theta\beta c x_3 x_4}{x_1 + x_2 + x_3 + x_4} - \mu x_4 = f_4.
\end{aligned}$$

Now choosing $\beta c = \tilde{\beta}$ as the bifurcation parameter and considering that at $R_0 = 1$, $\tilde{\beta} = \beta^* = \frac{(\mu + k)(\mu + r + \mu_d)}{(k + \mu q)}$, the Jacobian matrix of the system (1) evaluated at the disease free equilibrium is obtained as

$$H = \begin{pmatrix} -\mu & 0 & \beta^* & 0 \\ 0 & -(\mu + k) & (1-q)\beta^* & 0 \\ 0 & k & q\beta^* - (\mu + r + \mu_d) & 0 \\ 0 & 0 & r & -\mu \end{pmatrix}.$$

With $\tilde{\beta} = \beta^*$ the transformed system (1) has a simple eigenvalue with zero real part and all other eigenvalues are negative (i.e. has a hyperbolic equilibrium point). Thus, we can use the center manifold theory [1] to investigate dynamics of transformed system (1) near $\tilde{\beta} = \beta^*$. It is possible to obtain the right eigenvectors of $H(P_0)|_{\tilde{\beta}=\beta^*}$ which are denoted by $w = (w_1, w_2, w_3, w_4)^T$ where

$$w_1 = \frac{-\beta^* w_3}{\mu}, w_2 = \frac{(1-q)\beta^* w_3}{(\mu + k)}, w_4 = \frac{r w_3}{\mu}, w_3 = w_3 > 0.$$

Similarly we can obtain the left eigenvectors of $H(P_0)|_{\tilde{\beta}=\beta^*}$ denoted by $v = (v_1, v_2, v_3, v_4)$ where

$$v_1 = 0,\ v_2 = \frac{k v_3}{\mu + k},\ v_3 = v_3 > 0, v_4 = 0.$$

Now we proceed to obtain the associated bifurcation coefficients, a and b as described in Theorem 4.1 of [1]. For the purpose of clarity we restate Theorem 4.1 of [1].

Theorem 1 *(Castillo-Chavez and Song [1].) Consider the following general system of ordinary differential equations with a parameter* φ

$$\frac{dx}{dt} = f(x, \varphi),\ f : \mathbb{R}^n \times \mathbb{R} \to \mathbb{R} \text{ and } f \in \mathbb{C}(\mathbb{R}^n \times \mathbb{R}), \qquad (2)$$

where 0 is an equilibrium point of the system (that is, $f(0, \varphi) \equiv 0$ *for all* φ *and assume*

A1 : $A = D_x f(0,0) = \left(\frac{\partial f_i}{\partial x_j}(0,0)\right)$ *is the linearization matrix of the system 2 around the equilibrium 0 with* φ *evaluated at 0. Zero is a simple eigenvalue of A and other eigenvalues of A have negative real parts;*

A2 : *Matrix A has a right eigenvector w and a left eigenvector v (each corresponding to the zero eigenvalue).*

Let f_k be the kth component of f and

$$a = \sum_{k,i,j=1}^{n} v_k w_i w_j \frac{\partial^2 f_k}{\partial x_i \partial x_j}(0,0),$$
$$b = \sum_{k,i=1}^{n} v_k w_i \frac{\partial^2 f_k}{\partial x_i \partial \varphi}(0,0).$$

Then, the local dynamics of the system 2 around 0 are determined by the signs of a and b.

(i) $a > 0, b > 0$. *When $\varphi < 0$ with $|\varphi| \ll 1$, 0 is locally asymptotically stable and there exists a positive unstable equilibrium; when $0 < \varphi \ll 1$, 0 is unstable and there exists a negative, locally asymptotically stable equilibrium;*

(ii) $a < 0, b < 0$. *When $\varphi < 0$ with $|\varphi| \ll 1$ 0 is unstable; when $0 < \varphi \ll 1$, 0 is locally asymptotically stable equilibrium, and there exists a positive unstable equilibrium;*

(iii) $a > 0, b < 0$. *When $\varphi < 0$ with $|\varphi| \ll 1$ 0 is unstable, and there exists a locally asymptotically stable negative equilibrium; when $0 < \varphi \ll 1$, 0 is stable, and a positive unstable equilibrium appears;*

(iv) $a < 0, b > 0$. *when φ changes from negative to positive, 0 changes its stability from stable to unstable. Correspondingly a negative unstable equilibrium becomes positive and locally asymptotically stable.*

In particular, if $a > 0$ and $b > 0$, then a backward bifurcation occurs at $\varphi = 0$.

Computation of a.
The transformed model system (1) has the following non-vanishing partial derivatives of H evaluated at disease free equilibrium,

$$\frac{\partial^2 f_2}{\partial x_3 \partial x_2} = -\frac{2(1-q)\beta^*\mu}{\Lambda} - \frac{2p\beta^*\mu}{\Lambda}, \frac{\partial^2 f_2}{\partial x_3 \partial x_4} = -\frac{2(1-q)\beta^*\mu}{\Lambda} + \frac{2(1-\sigma)\theta\beta^*\mu}{\Lambda},$$
$$\frac{\partial^2 f_3}{\partial x_2 \partial x_3} = -\frac{-2q\beta^*\mu}{\Lambda} + \frac{2p\beta^*\mu}{\Lambda}, \frac{\partial^2 f_3}{\partial x_3 \partial x_4} = \frac{-2q\beta^*\mu}{\Lambda} + \frac{2\sigma\theta\beta^*\mu}{\Lambda},$$
$$\frac{\partial^2 f_2}{\partial x_3 \partial x_3} = \frac{-2(1-q)\beta^*\mu}{\Lambda}, \frac{\partial^2 f_3}{\partial x_3 \partial x_3} = \frac{-2q\beta^*\mu}{\Lambda}.$$

Hence,

$$\begin{aligned} a &= \sum_{k,i,j=1}^{4} v_k w_i w_j \frac{\partial^2 f_k(0,0)}{\partial x_i \partial x_j} \\ &= v_2 w_2 w_3 \frac{\partial^2 f_2(0,0)}{\partial x_2 \partial x_3} + v_2 w_3 w_4 \frac{\partial^2 f_2(0,0)}{\partial x_3 \partial x_4} + v_3 w_2 w_3 \frac{\partial^2 f_3(0,0)}{\partial x_2 \partial x_3} + v_3 w_3 w_4 \frac{\partial^2 f_3(0,0)}{\partial x_3 \partial x_4} \\ &\quad + v_2 w_3 w_3 \frac{\partial^2 f_2(0,0)}{\partial x_3 \partial x_3} + v_3 w_3 w_3 \frac{\partial^2 f_3(0,0)}{\partial x_3 \partial x_3} \\ &= \frac{2\mu^2(1-q)\beta^{*2} v_3 w_3^2}{\Lambda(\mu+k)} \left(p - (k+\mu q)\left(\frac{\mu(1-q)(\mu+r+\mu_d)+(\mu+r)(k+\mu q)}{\mu^2(1-q)(\mu+r+\mu_d)} \right) + \frac{r\theta(k+\sigma\mu)}{(\mu+r+\mu_d)\mu^2(1-q)} \right) \quad (3) \\ &= \frac{2\mu^2(1-q)\beta^{*2} v_3 w_3^2}{\Lambda(\mu+k)}(p - p_c). \end{aligned}$$

Computation of b.
The sign of bifurcation parameter b is associated with the following non-vanishing partial derivatives of F, also evaluated at disease free equilibrium;

$$\frac{\partial^2 f_2}{\partial x_3 \partial \beta^*} = (1-q), \frac{\partial^2 f_3}{\partial x_3 \partial \beta^*} = q.$$

Now

$$\begin{aligned} b &= \sum_{k,i=1}^{4} v_k w_i \frac{\partial^2 f_k(0,0)}{\partial x_i \partial \beta^*} \\ &= v_2 w_3 \frac{\partial^2 f_2(0,0)}{\partial x_3 \partial \beta^*} + v_3 w_3 \frac{\partial^2 f_3(0,0)}{\partial x_3 \partial \beta^*} \\ &= v_3 w_3 \frac{(k+\mu q)}{(\mu+k)} > 0. \end{aligned}$$

The eigenvectors v_3 and w_3 are positive. The bifurcation coefficient b is always positive. From Theorem 1 the model system (1) will exhibit backward bifurcation phenomena if the bifurcation coefficient a defined by (3) is positive. We can clearly see from (3) that the positivity of a is entirely dependent on the level of exogenous reinfection parameter p. This suggests existence of a bifurcation threshold below which backward bifurcation disappears and above which bi-stability phenomena occurs. After algebraic manipulation it can be shown that the bifurcation coefficient $a > 0$ whenever

$$\begin{aligned} p > p_c &= (k+\mu q)\left(\frac{\mu(1-q)(\mu+r+\mu_d) + (\mu+r)(k+\mu q)}{\mu^2(1-q)(\mu+r+\mu_d)} - \frac{r\theta(k+\sigma\mu)}{\mu^2(1-q)(\mu+r+\mu_d)}\right) \\ &= \frac{k+\mu q}{\mu(1-q)}\left(\frac{\mu(1-q)(\mu+r+\mu_d) + (\mu+r)(k+\mu q)}{\mu(\mu+r+\mu_d)} - F_r\right) \end{aligned}$$

where $F_r = \dfrac{r\theta(k+\sigma\mu)}{\mu(\mu+r+\mu_d)}$.

References

[1] Castillo-Chavez C, Song B. Dynamical models of tuberculosis and their applications. Math Biosci Eng. 2004;1(2):361–404.
