# Supplementary material for: Backward bifurcation and hysteresis in models of recurrent tuberculosis
Source: PLoS One. 2018 Mar 22;13(3):e0194256. doi: 10.1371/journal.pone.0194256 (PMC5863985; doi:10.1371/journal.pone.0194256)
Supplement: S3 Appendix — (PDF) [file pone.0194256.s003.pdf]

S3 Appendix. Equilibrium quantities expressed in terms of λ. The equilibrium quantities for model (1) in terms of λ are given as

$$S^* = \frac{\Lambda}{\lambda+\mu},$$

$$E^* = \frac{(1-q)\lambda\Lambda(\mu+\mu_d)(\theta\lambda+\mu)+(1-q)\lambda\Lambda r\mu+(1-\sigma)\theta r\Lambda\lambda^2}{(\mu+\mu_d)(\theta\lambda+\mu)(\lambda+\mu)(p\lambda+k+\mu)+r\theta\lambda\mu(1-\sigma)(\lambda+\mu)+r\mu(\lambda+\mu)(p\lambda+k+\mu)}, \tag{1}$$

$$I^* = \frac{q\lambda\Lambda(\theta\lambda+\mu)(p\lambda+k+\mu)+(1-q)\lambda\Lambda(\theta\lambda+\mu)(p\lambda+k)}{(\mu+\mu_d)(\theta\lambda+\mu)(\lambda+\mu)(p\lambda+k+\mu)+r\theta\lambda\mu(1-\sigma)(\lambda+\mu)+r\mu(\lambda+\mu)(p\lambda+k+\mu)},$$

$$R^* = \frac{rq\lambda\Lambda\mu+r\lambda\Lambda(p\lambda+k)}{(\mu+\mu_d)(\theta\lambda+\mu)(\lambda+\mu)(p\lambda+k+\mu)+r\theta\lambda\mu(1-\sigma)(\lambda+\mu)+r\mu(\lambda+\mu)(p\lambda+k+\mu)}.$$
