# Supplementary material for: Backward bifurcation and hysteresis in models of recurrent tuberculosis
Source: PLoS One. 2018 Mar 22;13(3):e0194256. doi: 10.1371/journal.pone.0194256 (PMC5863985; doi:10.1371/journal.pone.0194256)
Supplement: S4 Appendix — (PDF) [file pone.0194256.s004.pdf]

S4 Appendix. Descartes rule of signs.

Theorem 1 *[1] Let V be the number of variations in the sign of the coefficients $d_n, d_{n-1}, \cdots d_0$ (ignoring coefficients that are zero). Let n_p be the number of real positive zeros. Then*

(i) $n_p \leq V$,

(ii) $V - n_p$ *is an even integer.*

References

[1] Pinkert JR. An exact method for finding the roots of a complex polynomial. ACM Transactions on Mathematical Software (TOMS). 1976;2(4):351–363.
