# Supplementary material for: Backward bifurcation and hysteresis in models of recurrent tuberculosis
Source: PLoS One. 2018 Mar 22;13(3):e0194256. doi: 10.1371/journal.pone.0194256 (PMC5863985; doi:10.1371/journal.pone.0194256)
Supplement: S5 Appendix — (PDF) [file pone.0194256.s005.pdf]

S5 Appendix Further discussion on parameters.
With regard to parameter values, we note that the parameter p which accounts for level of reinfection, is often considered to lie in the interval $(0, 1)$ which takes into account that primary infection renders some degree of cross immunity to exogenous reinfection. A value of $p \in (1, \infty)$ suggests that initial TB infection increases the likelihood of active TB [1]. HIV-infected individuals, might be modelled with $p > 1$, given they have a compromised immune systems and enhanced susceptibility, as pointed out by Feng et al. [1]. In the original study by Feng et al. [1] the critical value p_c that could induce backward bifurcation phenomena was $p_c = 0.30$. They demonstrated both numerically and analytically that if $p = 0.4 > p_c = 0.3$, multiple equilibria could occur when $R_0 < 1$ thus fulfilling the classical requirement for backward bifurcation. Vinnicky and Fine [2] chose $p = 0.4$ but they never explored its role in inducing backward bifurcation. In several other studies such as Lopez [3] reinfection value was fixed to $p = 0.5$ while in Gomes et al. [4, 5] the baseline value for $p = 0.25$. Feng et al. [1] fixed θ to one with the justification that it will make mathematical analysis possible otherwise. In the study of [6] the critical value of θ was estimated to be about 5 which is unreasonably high, but again within the range given by Gomes et al. [5] $\theta \in 3.87[1.61, 7.79]$. In their numerical analysis Wang et al. [6] chose $\theta = 10$ which is large. Except where we use the assumption $p = 0$, in the proposed model with recurrent TB, we assume the conservative view that both $p, \theta \in (0, 1)$.

Regarding biological plausibility, it is observed in the Fig 4, where $p = 0.09 > p_c = 0.0658$, that $\sigma * \theta = 0 * 0 = 0 < q = 0.05$ yet there is a backward bifurcation in this biologically plausible range. Also in Fig 6 we have $\theta * \sigma = 0.3 * 0.05 = 0.015 < q = 0.05$ which again seems reasonable for the situation where recovered individuals have partial immunity against reinfection. Note also in Fig 6 again that $\theta * \sigma = 0.3 * 0.05 = 0.015 < q = 0.05$ in which there is no big difference between $\theta * \sigma$ and q. The only case where $\theta * \sigma$ is slightly higher than q is where there is assumption that latent TB individuals are not reinfected (have complete immunity $p = 0$ - see Eq (18) in the text). In this case it is found that backward bifurcation will only occur if recurrent TB due to reinfection is significantly high, i.e $\theta > \theta_c = 4.98 \approx 5$ (see equation 21 in the text) and $\sigma * \theta = 0.2 * 6 = 0.12$ which is about two-fold in comparison to q. While these values for θ are quite high, it is interesting to note that the θ values that induce backward bifurcations still lie within the estimated range $\theta \in 3.87[1.61, 7.79]$ [5].

The choice of per-capita recovery rate parameter $r = 2$ in Table 1 in the text corresponds to an average duration of infectiousness of six months i.e $r = 1/6$ months $= 1/0.5$year $= 2yr^-1$, as in the work of Feng et al. [1], and Gomes et al. [4, 7].

References

[1] Feng Z, Castillo-Chavez C, Capurro AF. A model for tuberculosis with exogenous reinfection. Theoretical population biology. 2000;57(3):235–247.

[2] Vynnycky E, Fine P. The natural history of tuberculosis: the implications of age-dependent risks of disease and the role of reinfection. Epidemiology and infection. 1997;119(02):183–201.

[3] Lopes JS, Rodrigues P, Pinho ST, Andrade RS, Duarte R, Gomes MGM. Interpreting measures of tuberculosis transmission: a case study on the Portuguese population. BMC infectious diseases. 2014;14(1):340–349.

[4] Gomes MGM, Rondrigues P, Hilker FM, Mantilla-Beniers NB, Muehlen M, Paulo AC, et al. Implications of partial immunity on the prospects for tuberculosis control by post-exposure interventions. Journal of theoretical biology. 2007;248(4):608–617.

[5] Gomes MGM, Águas R, Lopes JS, Nunes MC, Rebelo C, Rodrigues P, et al. How host heterogeneity governs tuberculosis reinfection? Proceedings Biological sciences. 2012 Feb: rspb20112712.

[6] Wang X, Feng Z, Aparicio J, Castillo-Chavez C. On the dynamics of reinfections: The case of Tuberculosis. In: International Symposium on Mathematical and Compuation Biology (ed. Rubem P. Mondaini), BIOMAT; 2009. p. 304–330.

[7] Gomes MGM, Franco AO, Gomes MC, Medley GF. The reinfection threshold promotes variability in tuberculosis epidemiology and vaccine efficacy. Proceedings of the Royal Society of London Series B: Biological Sciences. 2004;271(1539):617–623.
